# Insilico prediction and functional analysis of nonsynonymous SNPs in human *CTLA4* gene

**DOI:** 10.1038/s41598-022-24699-0

**Published:** 2022-11-28

**Authors:** Muhammad Irfan, Talha Iqbal, Sakina Hashmi, Uzma Ghani, Attya Bhatti

**Affiliations:** Healthcare Biotechnology, National University of Science and Technology, Islamabad H-12, 44000 Pakistan

**Keywords:** Biotechnology, Computational biology and bioinformatics, Molecular biology, Diseases, Health care

## Abstract

The *CTLA4* receptor is an immune checkpoint involved in the downregulation of T cells. Polymorphisms in this gene have been found to be associated with different diseases like rheumatoid arthritis, autosomal dominant immune dysregulation syndrome, juvenile idiopathic arthritis and autoimmune Addison's disease. Therefore, the identification of polymorphisms that have an effect on the structure and function of *CTLA4* gene is important. Here we identified the most damaging missense or non-synonymous SNPs (nsSNPs) that might be crucial for the structure and function of *CTLA4* using different bioinformatics tools. These in silico tools included SIFT, PROVEAN, PhD-SNP, PolyPhen-2 followed by MutPred2, I-Mutant 2.0 and ConSurf. The protein structures were predicted using Phyre2 and I-TASSER, while the gene–gene interactions were predicted by GeneMANIA and STRING. Our study identified three damaging missense SNPs rs1553657429, rs1559591863 and rs778534474 in coding region of *CTLA4* gene. Among these SNPs the rs1553657429 showed a loss of potential phosphorylation site and was found to be highly conserved. The prediction of gene–gene interaction showed the interaction of *CTlA4* with other genes and its importance in different pathways. This investigation of damaging nsSNPs can be considered in future while studying *CTLA4* related diseases and can be of great importance in precision medicine.

## Introduction

The human genome possesses various types of variation, but the amplest among these variations are single nucleotide polymorphisms (SNPs). There are roughly about 3–10 million SNPs which comprises almost 1% of the whole genome^1^. Non-synonymous SNP (nsSNPs) /missense SNPs residing in the coding region are very crucial and accounts for residual change which may have neutral or deleterious effect on protein^2,3^. These variations may account for some damaging effects i.e. protein structure destabilization, aberrant gene regulation^4^, alteration in protein hydrophobicity, proteins charge disturbance, change in protein geometry^5^, dynamics, translation, protein–protein interactions^6,7^ and loss of protein integrity^8^.

Missense mutations are responsible for almost 50% of the entire DNA mutations, associated with genetic diseases including inflammatory and autoimmune diseases either as causative or susceptibility factors^9–11^. Analysis of *PTPN22* identified missense mutation (R620W), which shows association with different autoimmune diseases including diabetes type 1^12^. Another study indicates missense mutation (Y402H) in *CFH*, increased the macular degeneration susceptibility^13^.

A significant number of studies have used insilico tools to predict the structural and functional impact of nsSNPs on different proteins. For instance, a study on *ABCA1* polymorphism revealed association with familial hypoalphalipoproteinemia and tangier disease^14^. Another study investigated the association of *CYP27B1* polymorphism with vitamin D deficiency^15^. Similarly, various other studies have used insilico tools to establish the role of nsSNPs in other human diseases such as mental disorders^16^ congenital cataracts^17^, rheumatoid arthritis^18^, steroid resistant nephrotic Syndrome^19^ and breast cancer^20^.

The computational analysis of damaging nsSNPs of *CTLA4* has not been conducted before. Human *CTLA4* is a receptor protein that belongs to immunoglobulin superfamily and is mainly expressed on activated T-cells. It functions as a negative regulator of T cells and competes with CD28 for binding with B7-1(CD-80) and B7-2 (CD-86) ligand present on the surface of Antigen Presenting Cells (APCs). It directly inhibits the T cells mediated immune response and blocks CD28 signaling which further leads to inhibition of T cells interaction with APCs^21,22^. The non-synonymous mutations in *CTLA4* gene might disturb its interaction with its ligands and can lead to autoimmune diseases and cancer. Therefore, studying the effect of nsSNPs on its structure and function is crucial for establishing its role in different diseases.

The current study investigates the structural and functional influence of nsSNPs on *CTLA4*. *CTLA4* (Cytotoxic T-lymphocyte-associated protein 4) also called CD152, encoded by *CTLA4* gene in human located at chromosome 2q33.2. It is a 223 amino acids long protein which belong to immunoglobulin family, having three exons encoding V like domain, hydrophobic putative transmembrane and putative cytoplasmic domain^23,24^. *CTLA4* act as an immune checkpoint, and downregulate T lymphocytes after CD80 or 86 attachment with it, evident from *CTLA4* deficient mouse^25^, and can lead to elevated level of blast cells and their infiltration to heart, lung, and pancreas tissue^26^. Different studies proposed association of *CTLA4* with rheumatoid arthritis (RA)^27^, autosomal dominant immune dysregulation syndrome^28^, juvenile idiopathic arthritis^29^ autoimmune Addison's disease (AAD)^30^ and Breast cancer^31^. Therefore, it is vital to analyze the potential damaging effect of nsSNPs on *CTLA4*. The most deleterious nsSNPs in *CTLA4* and their functional consequences have been predicted in this work by means of various in silico tools. The 3D model of wild type and its mutants have also been anticipated and the comparison is carried out to explore the diversion between wild and mutants resulting from nsSNPs. This is the first computational analysis of the *CTLA4* which predicts the deleterious effect of potential nsSNPs on structure, stability, protein–protein interaction and post translational modification of this protein using different publicly available computational tools. In future this study might be helpful for studying *CTLA4* associated diseases.

## Results

### Retrieved SNP

**Figure 1:**
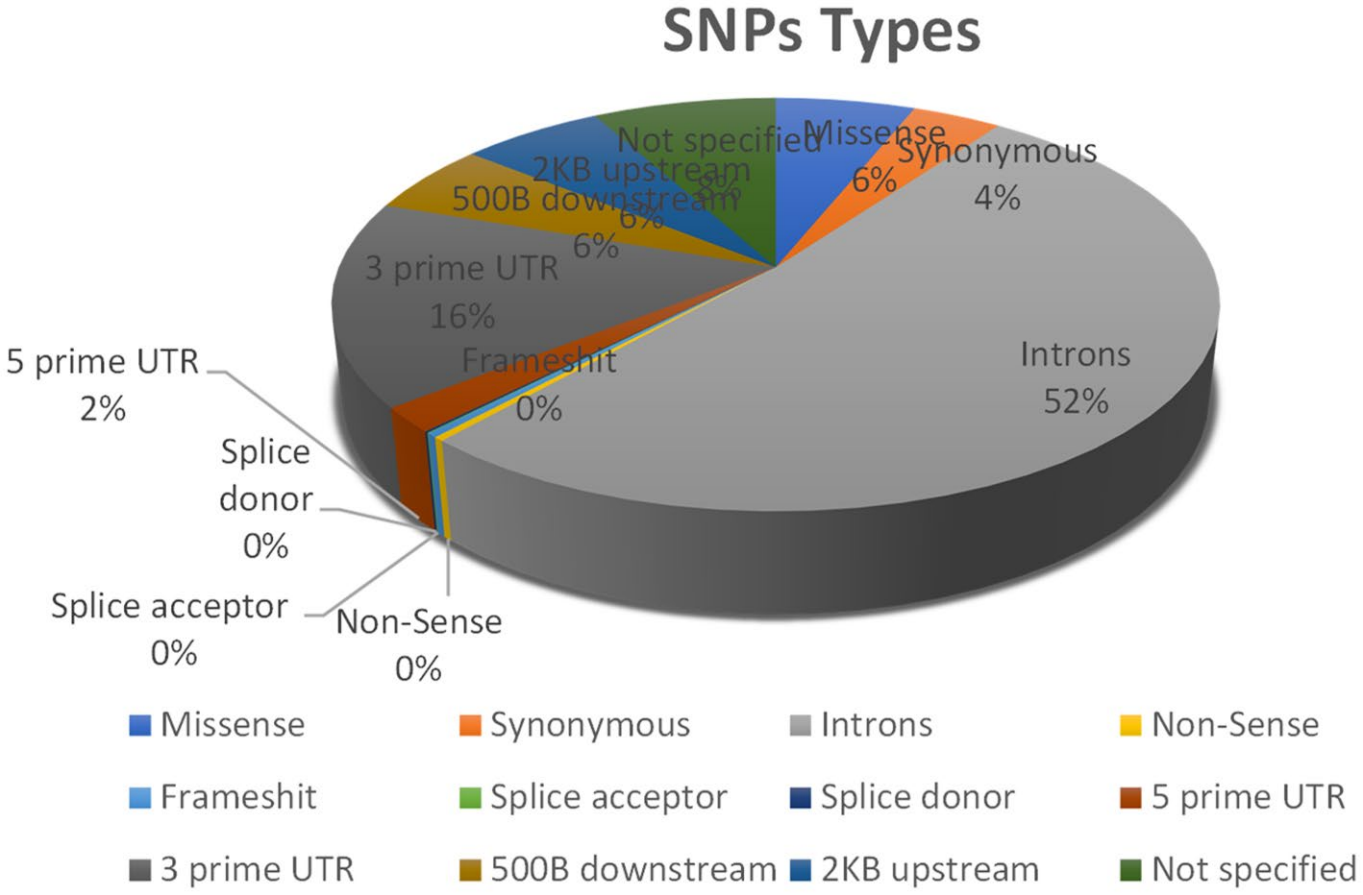
Percentage of all the SNPs in human CTLA4 gene.

The dbSNP provided a total of 1835 SNPs in *CTLA4* gene. Out of total SNPs, 945 were in intronic region, 111 were missense, 71 were found to be synonymous, 36 located in 5’UTR, while 294 were present in 3’UTR region and the remaining SNPs were (Nonsense = 6, Frameshift = 6, Splice acceptor = 1, Splice donor = 2, 500b downstream = 102, 2 Kb upstream = 113, Not specified = 146). Only the missense or nsSNPs were selected for further in silico analysis. The detailed information of all nsSNPs is given in Table S1 while Fig. 1 shows the graphical representation of percentage of all the SNPs.

### Damaging nsSNPs identified

**Figure 2:**
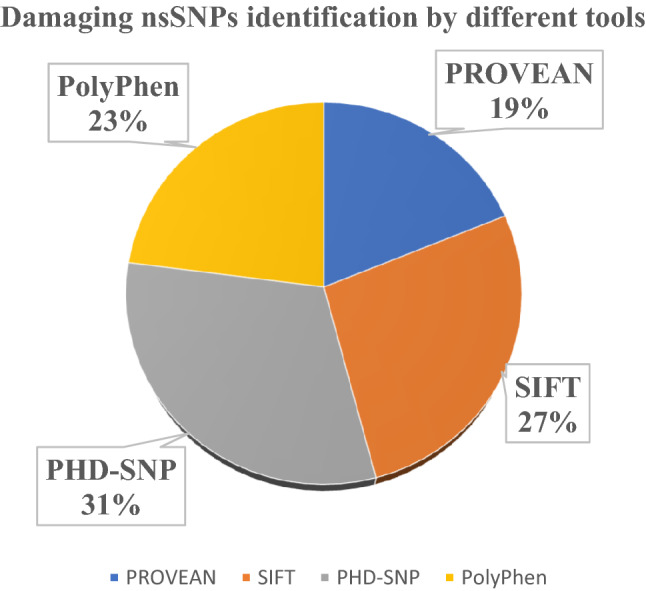
Percentage of potential damaging nsSNPs predicted through online computational tools.

All the nsSNPs obtained were subjected to four different computational tools to investigate their effect on the structure and function of CTLA4 protein. The different tools used were SIFT, PROVEAN, PolyPhen-2, and PhD‐SNP. A TI (Tolerance Index) threshold of 0.05 was taken for SIFT and the results having values less than the threshold were considered to be affected. SIFT identified 33 nsSNPs to be affected. For PROVEAN a value of − 2.5 cut off was considered as threshold and the nsSNPs having score below this value were considered deleterious. PROVEAN filtered a total of 23 nsSNPs to be deleterious. PhD‐SNP resulted in 38 SNPs to be diseased. The nsSNPs that were found damaging by all the three tools were further submitted to PolyPhen-2. Out of total SNPs submitted to above mentioned tools, 8 were found to be damaging by all the tools and were shortlisted for further analysis. The results of all the computational tools are summarized in Table S2, and Fig. 2.

### Structural and functional effects prediction by MutPred2

All the shortlisted 8 damaging nsSNPs were submitted to MutPred for predicting the impact of nsSNPs on CTLA4 protein structure and function. The probability scores of nsSNPs are given in the Table 1. The predictions made by MutPred2 include, loss of helix, gain of strand, Gain/loss of N-linked glycosylation and Sulfation, altered transmembrane protein, gain of Relative solvent accessibility, and altered ordered interface. The details of the above-mentioned predictions are given in the Table S3. These predictions suggest that many of the high risk nsSNPs effect the 3D structure of CTLA4 protein.

**Table 1:** MutPred2 P values of high risk nsSNPs identified in CTLA4.

Substitution	P-values	Substitution	P-Values
R70W	0.634	G118R	0.778
P137L	0.810	P138T	0.800
N145S	0.179	G146L	0.854
T147A	0.426	P209R	0.533

### Stability of protein

The function of a protein is associated with its stability that’s why it is important to identify the change in stability of protein due to nsSNPs. Mutant 2.0 predicted to what extent the damaging nsSNPs alter the stability of CTLA4 protein. The nsSNPs were submitted one by one and RI and DDG values were obtained. It was predicted that all of the damaging nsSNPs decrease the stability of CTLA4 protein except P137L. Three of the most damaging nsSNPs having highest RI values (P138T = 9, N145S = 9, T147A = 9) may be involved in causing greater damage to CTLA4 protein stability. The prediction of changes in stability of CTLA4 are given in Table 2.

**Table 2:** I-Mutant predicted CTLA4 protein stability due to deleterious nsSNPs.

SNP ID	Amino acid substitutions	Stability	RI	DDG value
rs606231422	R70W	Decrease	4	− 0.05
rs764089901	G118R	Decrease	7	− 0.02
rs1553657429	P137L	Increase	4	0.15
rs1553657430	P138T	Decrease	9	− 0.63
rs1204026047	N145S	Decrease	9	− 0.25
rs1466152724	T147A	Decrease	9	− 0.7
rs778534474	P209R	Decrease	8	− 0.49

### Conservation of amino acids

The damaging nsSNPs located in a highly conserved region can have more effect on protein structure and function as compared to damaging nsSNPs that are present in a region that is less conserved. The conservation profiles of CTLA4 amino acids were analyzed by ConSurf. The results provided by ConSurf are given in Fig. S1. According to predictions made by ConSurf, R70W and P137L were highly conserved and exposed, P138T and T147A were found to be highly conserved and buried, G118R was found to be buried while N145S, G146L and P209R were predicted to be exposed. The amino acids and their respective conservation scores are given in Table 3.

**Table 3:** Consurf results showing conservation scores of deleterious nsSNPs in CTLA4.

SNP ID	Residual Change	Conservation Score	Prediction
rs606231422	R70W	8	Highly conserved and exposed (f)
rs764089901	G118R	8	Buried
rs1553657429	P137L	9	Highly conserved and exposed (f)
rs1553657430	P138T	9	Highly conserved and buried (s)
rs1204026047	N145S	7	Exposed
rs1559591863	G146L	8	Exposed (f)
rs1466152724	T147A	9	Highly conserved and buried (s)
rs778534474	P209R	5	Exposed

### 3D modelling of CTLA4 and its mutants

**Figure 3:**
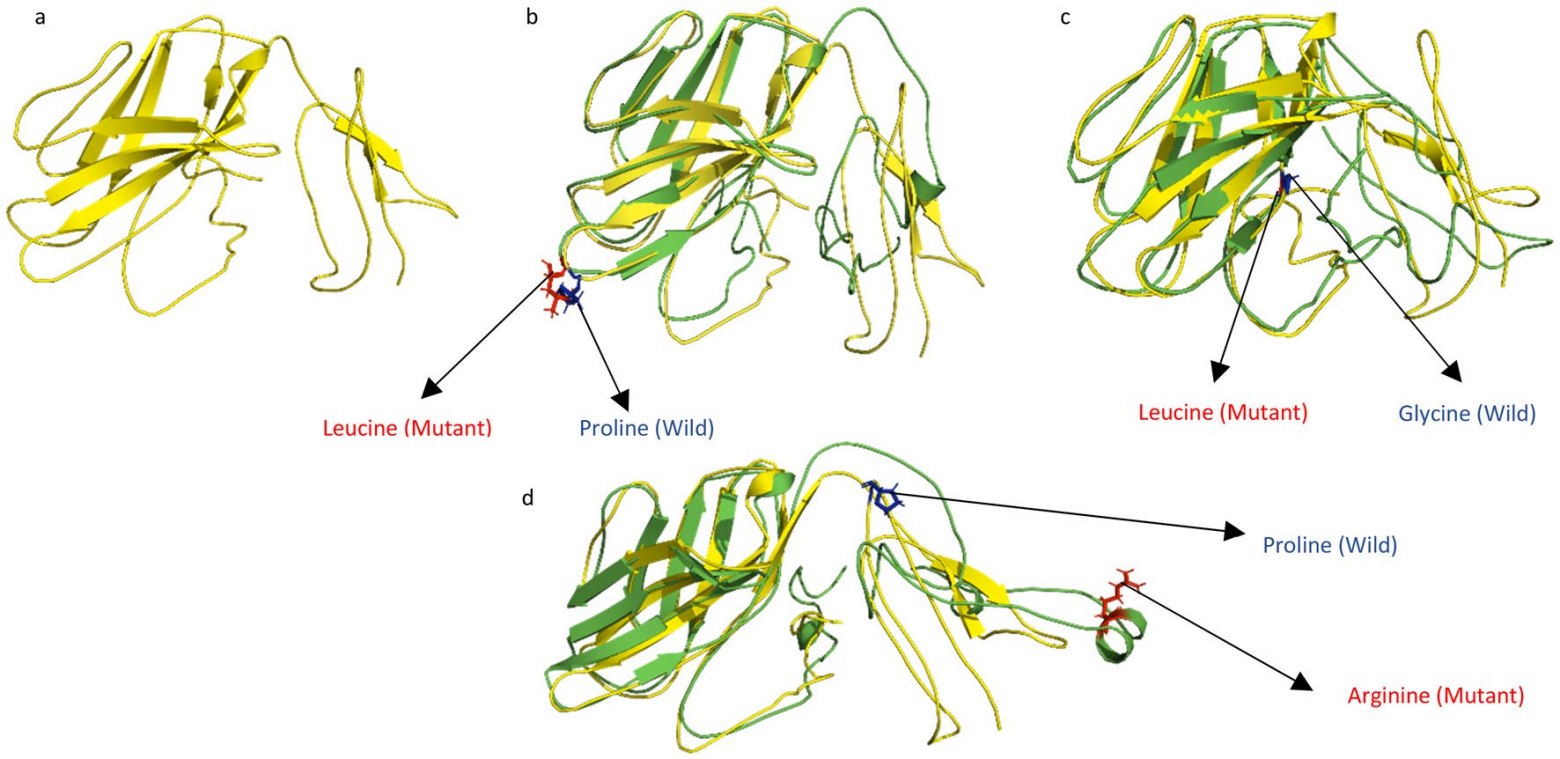
(**a**) Structure of Wild type CTLA4 protein. (**b**) Superimposed wild type CTLA4 protein and its mutant having Proline to Leucine mutation at position 137. (**c**) Superimposed wild type CTLA4 protein and mutant having Glycine to Leucine mutation at position 146. (**d**) Superimposed structure of wild type CTLA4 protein and mutant having mutation from Proline to Arginine at position 209.

The 3D structures of wild type *CTLA4* and 8 of its mutants were predicted by Phyre2. The Phyre2 results were incomplete as it predicted structure for only 118 amino acids (53% Coverage) out of 223 total amino acids. The wild type *CTLA4* and the mutants were then submitted to I-TASSER which is a more advance and reliable modeling tool. It predicted 5 models for CTLA4 protein and each of its mutant. The models with the highest C value were selected for further analysis. The protein models were also subjected to three different protein structure validation tools (MolProbity, ERRAT, and ProSA Web). For wild type CTLA4 the ERRAT calculated the overall quality factor as 57.672 whereas ProSA Web and MolProbity predicted − 3.49 Z-Score and 3.816 MolProbity score respectively which suggests that the protein structure was of good quality. The finalized 3 mutant models were also validated with the above-mentioned tools and the results showed Z-Score, overall quality factor and MolProbity Score for P137L (− 3.32, 58.13 and 3.815), G146L (− 3.48, 56.74 and 3.91) and P209R (− 3.4, 56.74 and 3.814) respectively. The protein structures were further refined with Galaxy Web. The predicted protein models were then compared using TM-Align to obtain TM-scores and RMSD values. The TM-score gives information about topological similarities between two proteins and RMSD values shows the average distance between backbone atoms of wild type and mutant proteins. The mutant with high RMSD value indicates greater deviation from its wild type. The mutant model for P137L (rs1553657429), P209R (rs778534474), G146L (rs1559591863), and P138T (rs1553657430) showed highest variation with RMSD values of 4.18, 3.73, 3.65, and 3.61 respectively. N145S and T147A showed RMSD values of 3.33 and 3.32 while R70W and G118R showed the lowest values of 3.0 and 2.49. Table 4 shows the TM-scores and RMSD values for all the models. The mutants having highest RMSD values (P137L, P209R, G146L) were selected and superimposed over wild type for further analysis using Chimera 1.14 shown in Fig. 3. The I-TASSER predicted structures were selected based on Confidence score (C-score). As per the reviewer/s comment the protein models were subjected to three different protein structure validation tools (MolProbity, ERRAT, and ProSA Web).

**Table 4:** TM-Align results showing TM-score and RMSD values of 8 mutants of CTLA4 protein.

SNP IDs	Residual change	TM-Score	RMSD values
rs606231422	R70W	0.83455	3.00
rs764089901	G118R	0.84663	2.49
rs1553657429	P137L	0.66498	4.18
rs1553657430	P138T	0.74708	3.61
rs1204026047	N145S	0.70885	3.33
rs1559591863	G146L	0.65038	3.65
rs1466152724	T147A	0.65591	3.32
rs778534474	P209R	0.67863	3.73

### PTM predictions

The results of Post translational modifications (PTMs) sites predicted by using different tools are discussed below.

#### Methylation

For the prediction of potential methylated sites in *CTLA4*, GPS‐MSP 3.0 was used, and no methylation sites were predicted.

#### Phosphorylation

The phosphorylated sites in *CTLA4* predicted by ModPred and NetPhos 3.1 are mentioned in Table S4. NetPhos predicted 20 residues and ModPred predicted 7 residues having phosphorylation potential. The amino acid residues that were found to be phosphorylated by both NetPhos and ModPred are Serine at position 62 and 194, Threonine at 197 and 207, and Tyrosine at position 60 and 201. The NetPhos 3.1 predicted that mutant P137L and P138T showed a loss of phosphorylation site at position 140, while T147A showed a loss of phosphorylation at 147 and gain at position 150.

#### Glycosylation

Potential glycosylated sites were found by N-Glyde and NetOGlyc4.0. N-Glyde predicted 2 sites 113 and 145 with scores 0.732072 and 0.9271713 respectively, to be N glycosylated while NetOGlyc4.0 predicted no site to be glycosylated. N-Glyde also predicted that mutant N145S and T147A showed loss of N-glycosylation at position 145. The results are given in Table S5.

#### Ubiquitylation

UbPred predicted 3 residues in CTLA4 capable of ubiquitination while BDM-PUB predicted 5 residues to get ubiquitinated and mutant R70W showed loss of ubiquitination at position 65. None of these ubiquitylation sites predicted were at deleterious SNPs regions. The results obtained from BDM-PUB and UbPred are given in Table S6.

#### Gene–gene interaction

**Figure 4:**
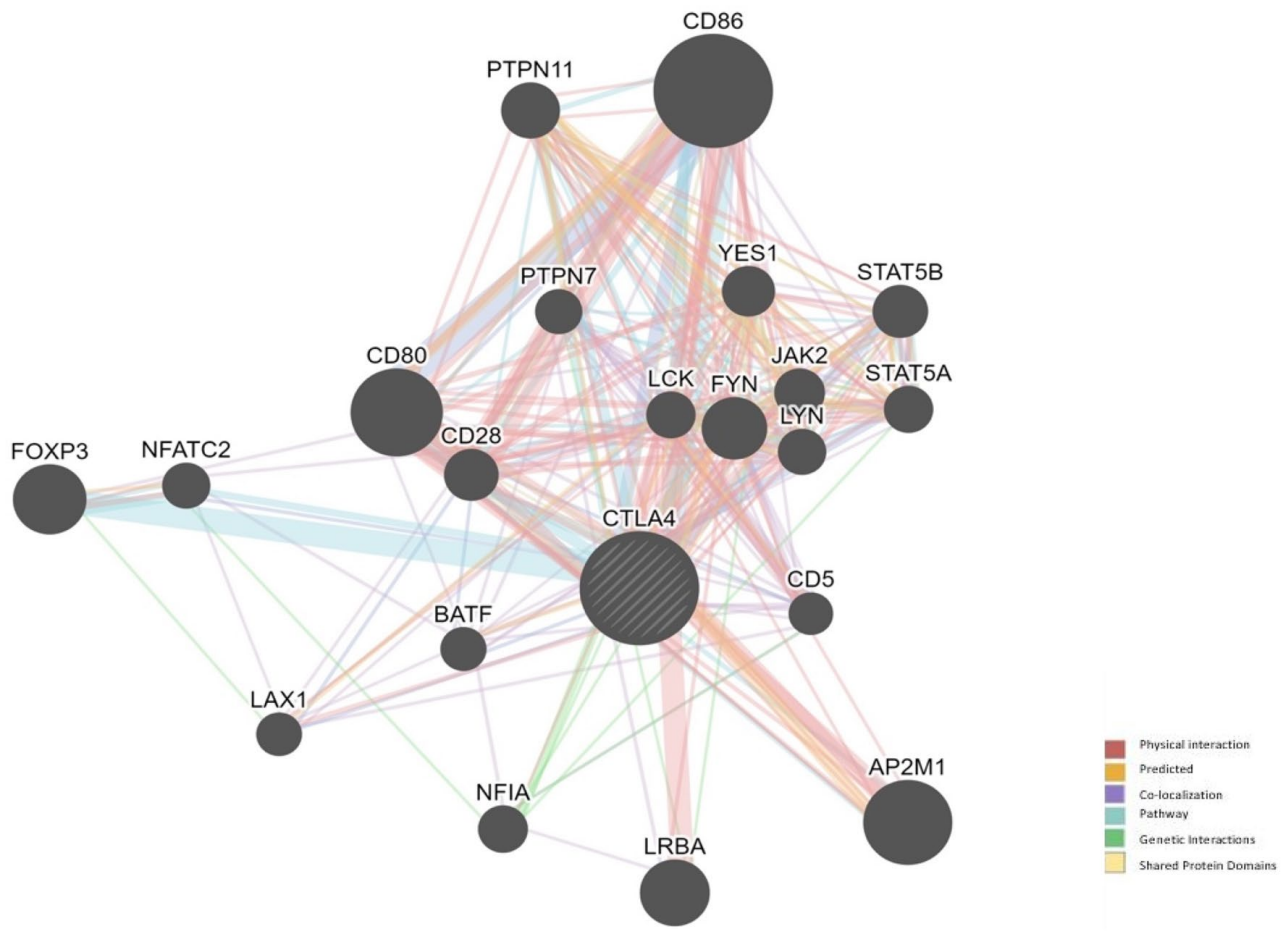
Gene–Gene Interaction of CTLA4 predicted by GeneMANIA. The CTLA4 shows main physical interaction with CD80, CD86, and AP2M1.

**Figure 5:**
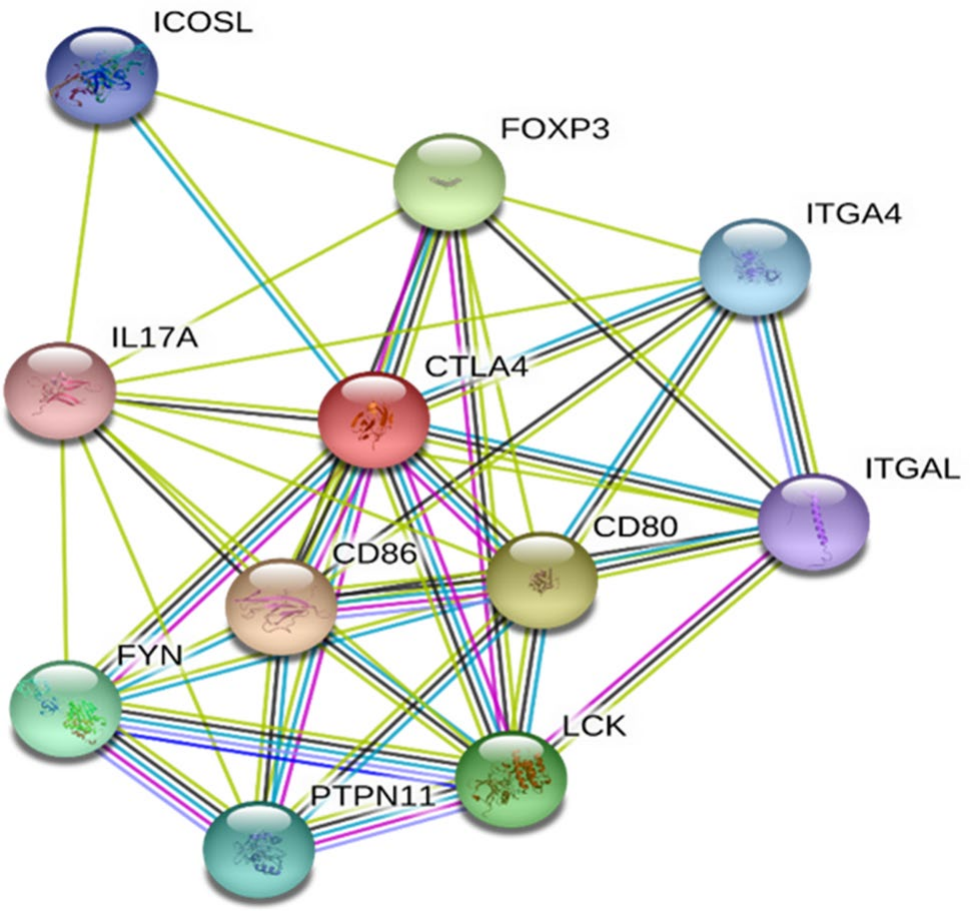
Gene–Gene interaction of CTLA4 predicted by STRING showing major interaction with CD80, CD86, and FOXP3.

For the prediction of interaction of *CTLA4* with other genes inside the cell GeneMANIA and STRING were used. Results obtained from STRING are given in Table S7. The GeneMANIA predicted physical interaction of *CTLA4* with *CD80, CD86, AP2M1, JAK2, STAT5A, STAT5B, PTPN11* and *FYN*. The genes that were predicted to be co-expressed with *CTLA4* are CD5, CXCL9, GPR132, CD200, CTSZ, JAK2 and FYN. In pathways it showed relation with *CD86, PTPN11, CD80, FOXP3, CD28, PTPN7, PTPN6*, and *NFATC2*. Co-localization was found with *STAT5B, CD86, FYN, STAT5A, PLA2G2D, CD28, GPR132, CD4 CXCL9, CTSZ, PTPN6* and *NFATC2*. The proteins that were predicted to share domain with *CTLA4* are *CD28, CD80* and *CD86*. Predictions made by GeneMania and STRING are given in Figs. 4, and 5 respectively.

## Discussion

The function of a protein is determined by the tertiary structure and therefore any modification in the amino acid sequence of that protein can have the potential to change the structure of protein and lead to disease. Bioinformatics analysis gives us the opportunity to predict the structural and functional effects of single nucleotide polymorphisms on a protein using different tools and algorithms. However, the sophistication of these algorithms is completely dependent on raw experimental data. The inauthentic and inaccurate raw data can lead to incorrect downstream structural and functional analysis. Therefore, it is suggested to use multiple tools and draw a consensus by comparing the results obtained from these tools. Furthermore, the bioinformatics results should be validated in the laboratory through different in-vitro and in-vivo experiments.

Different studies have investigated the role of *CTLA4* polymorphism with various diseases. The association of *CTLA4* polymorphism has been established with various autoimmune diseases like rheumatoid arthritis^30,32,33^, type 1 diabetes^34,35^, and multiple sclerosis^36,37^ and also different cancers such as breast cancer^38,39^, colorectal cancer^40,41^, lung cancer^42,43^, and cervical cancer^44,45^.

In the present study a total of 1835 SNPs were obtained from dbSNP out of which 111 non-synonymous or missense SNPs were subjected to different in silico tools including SIFT, PROVEAN, Polyphen2, and PhD‐SNP. These in silico tools predicted 8 SNPs to be damaging while other were found to be neutral. The damaging SNPs were subjected to further computational analysis to investigate their effect on protein structure and function. All these SNPs decreased protein stability as predicted by Mutant 22.0 except P137L. The amino acids that are directly involved in biological processes tend to be more conserved and thus changes in these amino acids will significantly affect the function of protein (Miller and Kumar, 2001). The conservation analysis for *CTLA4* revealed that rs606231422 at position R70W and rs1553657429 at position P137L were found highly conserved and exposed while rs1553657430 and rs1466152724 at positions P138T and T147A respectively were found to be highly conserved and buried. The rest of the SNPs were only found buried or exposed and not very conserved. The mutation in buried resides of protein can affect the structural integrity of the protein whereas the polymorphism in exposed resides may alter the protein function^46^. The structure of *CTLA4* and its mutants were predicted via I-TASSER. The nsSNPs directly influence the structure and hence function of a protein therefore, the effect of nsSNPs on structure of *CTLA4* was assessed^47^. It was observed that the predicted mutant structures of *CTLA4* have significantly distinguished RMSD values than the wild type and may compromise the structural integrity of the protein^47^.

For PTMs predictions of our protein different in silico tools were used. Phosphorylation is an important PTM which can activate or deactivate a protein by changing its structural conformation. The NetPhos result showed that mutant P137L and P138T have lost a phosphorylation site at position 40 and T147A lost a phosphorylation site at position 147 and gained phosphorylation site at position 150. As T147 is one of the most damaging nsSNP predicted in this study and was also found to be highly conserved and buried, that’s why a loss of phosphorylation at this site can be very significant for protein structure and function. The mutations that leads to the abolishing of a phosphorylation site can cause a direct deleterious effect on protein^48^. Similarly, N-Glyde showed that mutant N145S and T147A resulted in loss of N-glycosylation at position 145 which is the site of another most damaging SNP that’s why a loss of glycosylation at position 145 is important.

The gene–gene interaction was performed to identify the interacting partners of CTLA4 protein. The mutation analysis performed in the present study is important in this regard as mutation especially in ligand binding domains and motifs can disrupt the interaction of CTLA4 with its interacting proteins such as CD80, and CD86 which can lead to various disease conditions.

The domain analysis of the CTLA4 protein was performed to check the location of the predicted mutations in different domains of CTLA4. It was found that two of the predicted mutations (P137L, and G146L) were found to be in Immunoglobulin V-set domain, while the third mutation site (P209R) was in cytoplasmic domain. The mutations in the Immunoglobulin V-set domain can alter the binding affinity of CTLA4 with CD80 and CD80 that are involve in the negative regulation of T-cells whereas, the polymorphism lied in the cytoplasmic domain may affect the binding of multiple proteins i.e., PI3K, lipid kinase phosphatidylinositol 3-kinase (PI3K), the phosphatases SH2 domain containing protein tyrosine phosphatases (SHP-2), the serine threonine phosphatase PP2A and clathrin adaptor proteins activator protein1 (AP-1) and AP-2 may results in cancer development^49,50^.

The three nsSNPs in *CTLA4* identified in the current study are unique and their association with human diseases have not been assessed in wet lab experiments. It is evident from this insilico analysis that these nsSNPs have resulted in lower stability of *CTLA4* protein in comparison with their wild type protein. Moreover, the mutant proteins deviated in structure and showed loss of potential PTMs sites. It has been established that *CTLA4* is a negative regulator of T cells and inhibit immune responses by interacting with CD80 and CD86^22^. The proper interaction of *CTLA4* with its ligand is very crucial for its immune inhibitory function. Analysis of mutations especially in the ligand binding domain of CTLA4 protein can disrupt its interaction with ligands which can lead to various autoimmune diseases and also cancer.

The current study predicted high-risk SNPs in *CTLA4* which can potentially disrupt ligand-receptor interactions. However, further in-vitro and in-vivo studies are required to investigate and establish the role of these nsSNPs in different diseases. Moreover, Molecular Dynamics (MD) simulation analysis of the proteins predicted is required to study the stability and structural flexibility of predicted wild-type and mutant proteins in dynamic environment.

## Methods

**Figure 6:**
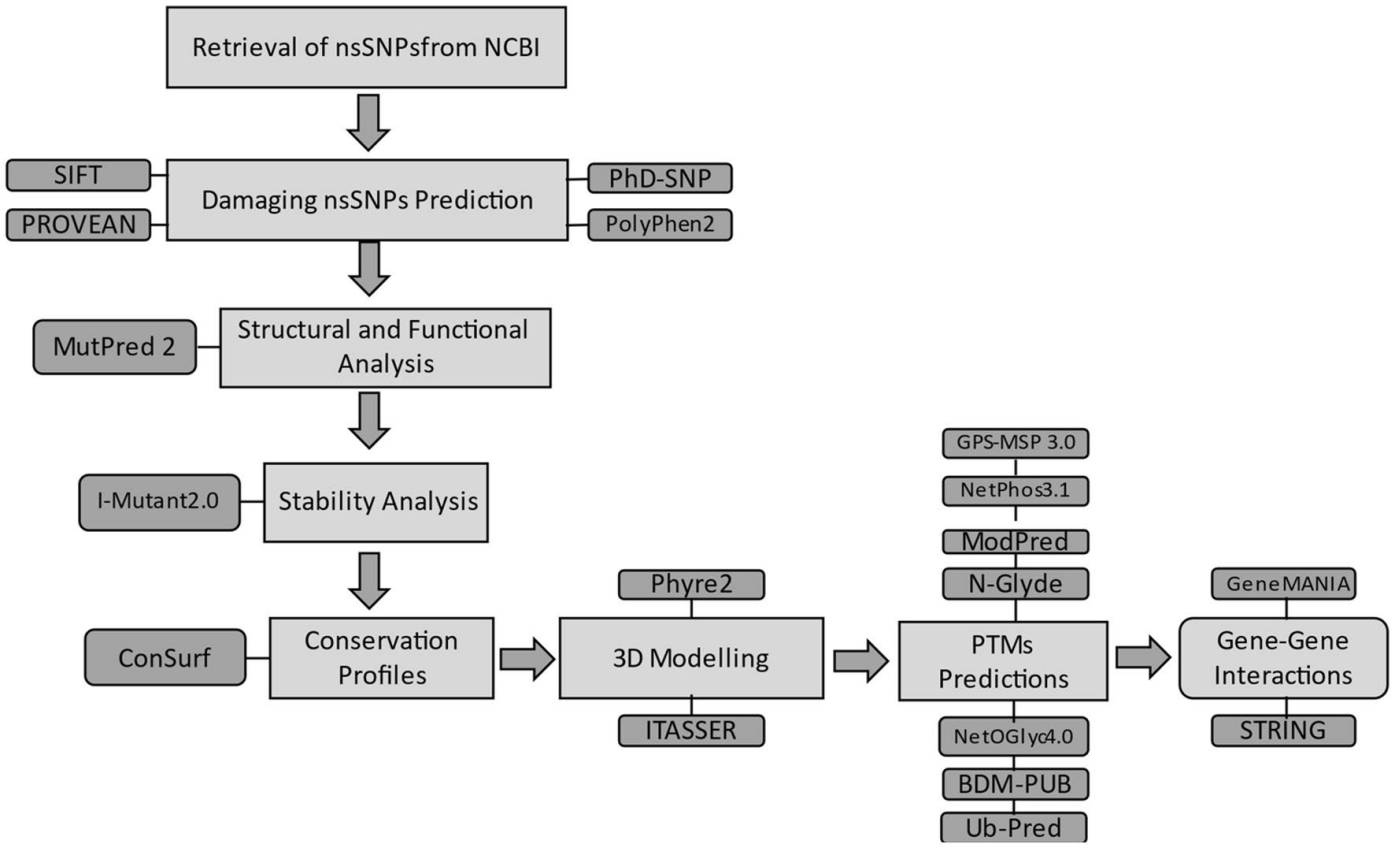
Flowchart representing the methodology of the study.

A schematic flowchart of complete methodology is given in Fig. 6.

### SNP data mining

The NCBI dbSNP (https://www.ncbi.nlm.nih.gov/snp/) (accessed: 20 April,2020) database was used to retrieve all the SNPs of *CTLA4* gene. The identification number (rsIDs) of nsSNPs were obtained from NCBI and protein sequence of *CTLA4* in FASTA format was retrieved from UniProt (https://www.uniprot.org) Only the missense or non-synonymous SNPs (nsSNPs) were selected for further in silico study.

### Identification of high risk nsSNPs

After retrieving the nsSNPs and protein sequence, the functionally damaging nsSNPs were predicted using different in silico tools including, SIFT (Sorting Intolerant From Tolerant)^51^, PROVEAN (Protein Variation Effect Analyzer)^52^, and PhD‐SNP (Predictor of human Deleterious SNP)^53^. The damaging nsSNPs found by these in silico tools were then submitted to PolyPhen2 (Polymorphism Phenotyping 2)^54^. The protein sequence in FASTA format and details of amino acids substitutions were used as input data for PolyPhen2.

### Prediction of nsSNPs effects on structure and function of CTLA4 protein

To analyze the structural and functional effect of nsSNPs on CTLA4 protein, MutPred2 was used^55^. It is a web application which predicts the pathogenicity of amino acid change in a protein. The sequence of CTLA4 protein was submitted in FASTA format to MutPred 2 along with the information of amino acid substitutions. The *p-* value less than 0.05 (*p* < 0.05) was taken as “Confident” and less than 0.01 (*p* < 0.01) as “Very Confident”.

### Prediction of protein stability

To study the influence of all the damaging nsSNPs on the stability of CTLA4 protein, I‐Mutant 2.0 was used. It is an online tool based on support vector machines (SVM) which predicts the extent to which a mutation affects a protein stability. The protein sequence of *CTLA4* gene was submitted at 25 °C with pH = 7.0. It gives result in the form of RI (Reliability index) with the values ranging from 0 to 10 (0 showing lowest and 10 showing highest reliability)^56^.

### Prediction of evolutionary conservation of CTLA4

ConSurf server was used for the prediction of the effect of nsSNPs on amino acids that are evolutionary conserved in CTLA4. ConSurf predicts the conserved amino acids in each protein by analyzing phylogenetic relation among homologous sequences using an empirical Bayesian inference and gives conservation scores ranging from 1 to 9^57^. FASTA sequence of CTLA4 was submitted as input option. The nsNSPs that were identified as highly conserved were further analyzed.

### Protein 3D structure prediction

The 3D models for native and mutant (R70W, G118R, P137L, P138T, N145S, G146L, T147A, and P209R) CTLA4 gene were predicted using Phyre2 . It is an online tool which predicts 3D models for protein based on principles of homology modeling^58^. The wild type CTLA4 and selected mutant proteins were then submitted to I-TASSER^59^ for remodeling. The I-TASSER predicted top five protein structures for wild type and all the mutants using fold recognition or threading approach. Among the top 5 predicted models the best models were selected for further study. After that the wild type and all the mutant models were compared using an online structure alignment tool called TM-Align which provides TM scores (Template Modelling score) and RMSD (root‐mean‐square deviation) values. The values of TM score ranges from 0 to 1, and 1 means the two proteins are perfectly matching. The higher RMSD means high structure variations between mutant and wild‐type and vice versa^60^. Three mutants with high RMSD values were selected and further analyzed using Chimera V1.14^61^.

### Prediction of potential PTM sites

Post translational modifications (PTMs) are very important for the structure, folding and proper function of proteins. Potential PTMs sites in CTLA4 protein and the gain/loss of PTMs sites in all the mutants due to nsSNPS were identified using several in silico tools. The sites where methylation occur in CTLA4 protein were predicted using GPS‐MSP^62^. For the prediction of phosphorylation at serine, threonine and tyrosine sites in CTLA4, ModPred^63^ and NetPhos3.1 was used. For NetPhos 3.1 the threshold was set to 0.5 and the amino acids having values higher than the threshold were predicted to be phosphorylated^64^. The potential glycosylation sites in CTLA4 were predicted by NetOglyc4.0^65^ and N-Glyde. For N-Glyde the residues with prediction score higher than 0.6 were predicted to have glycosylation potential^66^. BDM‐PUB, and UbPred were used for predicting Ubiquitylation sites in CTLA4. UbPred showed lysine residues having ubiquitylation potential with score equal to or higher than the threshold (0.62)^67^. For BDM-PUB balanced cut-off was selected^68^.

### Interaction of CTLA4 with other proteins

A protein interacts with many other proteins inside the cell and this interaction is important for the function and regulation of protein. The functional interaction of CTLA4 with other proteins inside the cells was predicted using GeneMANIA and STRING (accessed: 16 June 2020). The GeneMANIA use different parameters including genetic and protein interaction, co-expression, co-localization, pathways and protein domain similarities to predicts the interaction of input gene with many other genes^69^. STRING uses its database of 24′584′628 proteins from 5′090 organisms and predicts protein protein–protein interaction networks either through direct or indirect association among proteins^70^. The terms CTLA4 and Homo sapiens were searched as input options for both the tools.

## Conclusion

This study identified 3 major high risk nsSNPs, rs1553657429 (P137L), rs1559591863 (G146L), rs778534474 (P209R) within the coding region of *CTLA4* gene. They may have a major role in diseases associated with *CTLA4* gene as they are involved in decreasing the stability of protein and loss of potential phosphorylation site. The mutants possessing these nsSNPs showed deviation in structure from wild type CTLA4 protein. These nsSNPs can be significance for therapeutic strategies and personalized medicine and can be used for further experimental investigations to study the role of these nsSNPs in pathogenesis of related diseases.

## Supplementary Information

Supplementary Information.

## Data Availability

The datasets used and/or analyzed during the current study are available from the corresponding author on reasonable request.

## References

[1] Wright, A. F. Genetic variation: polymorphisms and mutations. *e LS* (2001).

[2] Collins FS, Guyer MS, Charkravarti A (1997). Variations on a theme: Cataloging human DNA sequence variation. Science.

[3] Capriotti E, Altman RB (2011). Improving the prediction of disease-related variants using protein three-dimensional structure. BMC Bioinform..

[4] Barroso I (1999). Dominant negative mutations in human PPARγ associated with severe insulin resistance, diabetes mellitus and hypertension. Nature.

[5] Petukh M, Kucukkal TG, Alexov E (2015). On human disease-causing amino acid variants: Statistical study of sequence and structural patterns. Hum. Mutat..

[6] Chasman D, Adams RM (2001). Predicting the functional consequences of non-synonymous single nucleotide polymorphisms: structure-based assessment of amino acid variation. J. Mol. Biol..

[7] Kucukkal TG, Petukh M, Li L, Alexov E (2015). Structural and physico-chemical effects of disease and non-disease nsSNPs on proteins. Curr. Opin. Struct. Biol..

[8] Thomas R (1999). Identification of mutations in the repeated part of the autosomal dominant polycystic kidney disease type 1 gene, PKD1, by long-range PCR. Am. J. Hum. Genet..

[9] Krawczak M (2000). Human gene mutation database: A biomedical information and research resource. Hum. Mutat..

[10] Santana-de Anda K, Gómez-Martín D, Díaz-Zamudio M, Alcocer-Varela J (2011). Interferon regulatory factors: beyond the antiviral response and their link to the development of autoimmune pathology. Autoimmun. Rev..

[11] Begovich AB (2004). A missense single-nucleotide polymorphism in a gene encoding a protein tyrosine phosphatase (PTPN22) is associated with rheumatoid arthritis. Am. J. Hum. Genet..

[12] Criswell LA (2005). Analysis of families in the multiple autoimmune disease genetics consortium (MADGC) collection: The PTPN22 620W allele associates with multiple autoimmune phenotypes. Am. J. Hum. Genet..

[13] Zareparsi S (2005). Strong association of the Y402H variant in complement factor H at 1q32 with susceptibility to age-related macular degeneration. Am. J. Hum. Genet..

[14] Marín-Martín FR, Soler-Rivas C, Martín-Hernández R, Rodriguez-Casado A (2014). A comprehensive in silico analysis of the functional and structural impact of nonsynonymous SNPs in the ABCA1 transporter gene. Cholesterol.

[15] Rotimi SO, Peter O, Oguntade O, Rotimi OA (2018). In silico analysis of the functional non-synonymous single nucleotide polymorphisms in the human CYP27B1 gene. Egypt. J. Med. Hum. Genet..

[16] Desai M, Chauhan J (2016). In silico analysis of nsSNPs in human methyl CpG binding protein 2. Meta Gene.

[17] Zhang M, Huang C, Wang Z, Lv H, Li X (2020). In silico analysis of non-synonymous single nucleotide polymorphisms (nsSNPs) in the human GJA3 gene associated with congenital cataract. BMC Mol. Cell Biol..

[18] Akhtar M (2021). Characterization of rheumatoid arthritis risk-associated SNPs and identification of novel therapeutic sites using an in-silico approach. Biology.

[19] Joshi BB (2015). In silico analysis of functional nsSNPs in human TRPC6 gene associated with steroid resistant nephrotic syndrome. Gene.

[20] Rajasekaran R, Sudandiradoss C, Doss CG, Sethumadhavan R (2007). Identification and in silico analysis of functional SNPs of the BRCA1 gene. Genomics.

[21] Zhao Y (2018). Evolving roles for targeting CTLA-4 in cancer immunotherapy. Cell Physiol. Biochem..

[22] Tai X (2012). Basis of CTLA-4 function in regulatory and conventional CD4+ T cells. Blood.

[23] Brunet JF (1987). A new member of the immunoglobulin superfamily–CTLA-4. Nature.

[24] Dariavach P, Mattéi MG, Golstein P, Lefranc MP (1988). Human Ig superfamily CTLA-4 gene: Chromosomal localization and identity of protein sequence between murine and human CTLA-4 cytoplasmic domains. Eur. J. Immunol..

[25] Vandenborre K (1999). Interaction of CTLA-4 (CD152) with CD80 or CD86 inhibits human T-cell activation. Immunology.

[26] Waterhouse P (1995). Lymphoproliferative disorders with early lethality in mice deficient in Ctla-4. Science.

[27] Plenge RM (2005). Replication of putative candidate-gene associations with rheumatoid arthritis in >4,000 samples from North America and Sweden: Association of susceptibility with PTPN22, CTLA4, and PADI4. Am. J. Hum. Genet..

[28] Schubert D (2014). Autosomal dominant immune dysregulation syndrome in humans with CTLA4 mutations. Nat. Med..

[29] Zhang L, Liang H, Guan H, Liu H (2015). Study of the association between CD28/CTLA-4 expression and disease activity in juvenile idiopathic arthritis. Exp. Ther. Med..

[30] Vaidya B (2000). Association analysis of the cytotoxic T lymphocyte antigen-4 (CTLA-4) and autoimmune regulator-1 (AIRE-1) genes in sporadic autoimmune Addison's disease. J. Clin. Endocrinol. Metab..

[31] Goske M (2017). CTLA-4 genetic variants (rs11571317 and rs3087243): Role in susceptibility and progression of breast cancer. World J. Oncol..

[32] Vaidya B (2002). An association between the CTLA4 exon 1 polymorphism and early rheumatoid arthritis with autoimmune endocrinopathies. Rheumatology.

[33] Yanagawa T, Gomi K, Nakao E-I, Inada S (2000). CTLA-4 gene polymorphism in Japanese patients with rheumatoid arthritis. Thyroid.

[34] Bouqbis L (2003). Association of the CTLA4 promoter region (− 1661G allele) with type 1 diabetes in the South Moroccan population. Genes Immun..

[35] Borysewicz-Sańczyk H (2020). Genetic association study of IL2RA, IFIH1, and CTLA-4 polymorphisms with autoimmune thyroid diseases and type 1 diabetes. Front. Pediatr..

[36] Yousefipour G, Erfani N, Momtahan M, Moghaddasi H, Ghaderi A (2009). JCTLA4 exon 1 and promoter polymorphisms in patients with multiple sclerosis. Acta Neurol. Scand..

[37] Alizadeh M (2003). Genetic interaction of CTLA-4 with HLA-DR15 in multiple sclerosis patients. Ann. Neurol..

[38] Wang L (2007). Association of CTLA-4 gene polymorphisms with sporadic breast cancer in Chinese Han population. BMC Cancer.

[39] Dai Z (2017). CTLA-4 polymorphisms associate with breast cancer susceptibility in Asians: a meta-analysis. PeerJ.

[40] Zou C (2018). CTLA4 tagging polymorphisms and risk of colorectal cancer: a case–control study involving 2,306 subjects. Onco Targets Ther..

[41] Hadinia A (2007). CTLA-4 gene promoter and exon 1 polymorphisms in Iranian patients with gastric and colorectal cancers. J. Gastroenterol. Hepatol..

[42] Antczak A (2013). Ctla-4 expression and polymorphisms in lung tissue of patients with diagnosed non-small-cell lung cancer. BioMed Res. Int..

[43] Khaghanzadeh N, Erfani N, Ghayumi MA, Ghaderi A (2010). CTLA4 gene variations and haplotypes in patients with lung cancer. Cancer Genet. Cytogenet..

[44] Gokhale P, Kerkar S, Tongaonkar H, Salvi V, Mania-Pramanik JJ (2013). CTLA-4 gene polymorphism at position+ 49 A> G in exon 1: a risk factor for cervical cancer in Indian women. Int. J. Immunol. Genet..

[45] Rahimifar S, Erfani N, Sarraf Z, Ghaderi A (2010). ctla-4 gene variations may influence cervical cancer susceptibility. Gynecol. Oncol..

[46] Gong H (2017). Improving prediction of burial state of residues by exploiting correlation among residues. BMC Bioinform..

[47] Shah H (2022). Impact of deleterious missense PRKCI variants on structural and functional dynamics of protein. Sci. Rep..

[48] Niu T (2016). Identification of IDUA and WNT16 phosphorylation-related non-synonymous polymorphisms for bone mineral density in meta-analyses of genome-wide association studies. J. Bone Miner. Res..

[49] Schneider H, Prasad K, Shoelson SE, Rudd CE (1995). CTLA-4 binding to the lipid kinase phosphatidylinositol 3-kinase in T cells. J. Exp. Med..

[50] Lee K-M (1998). Molecular basis of T cell inactivation by CTLA-4. Science.

[51] Sim N-L (2012). SIFT web server: Predicting effects of amino acid substitutions on proteins. Nucleic Acids Res..

[52] Choi Y, Sims GE, Murphy S, Miller JR, Chan AP (2012). Predicting the functional effect of amino acid substitutions and indels. PLoS ONE.

[53] Capriotti E, Calabrese R, Casadio R (2006). Predicting the insurgence of human genetic diseases associated to single point protein mutations with support vector machines and evolutionary information. Bioinformatics.

[54] Adzhubei IA (2010). A method and server for predicting damaging missense mutations. Nat. Methods.

[55] Pejaver V (2017). MutPred2: Inferring the molecular and phenotypic impact of amino acid variants. bioRxiv.

[56] Capriotti E, Fariselli P, Casadio R (2005). I-Mutant2.0: Predicting stability changes upon mutation from the protein sequence or structure. Nucleic Acids Res..

[57] Ashkenazy H, Erez E, Martz E, Pupko T, Ben-Tal N (2010). ConSurf 2010: calculating evolutionary conservation in sequence and structure of proteins and nucleic acids. Nucleic Acids Res..

[58] Kelley LA, Mezulis S, Yates CM, Wass MN, Sternberg MJ (2015). The Phyre2 web portal for protein modeling, prediction and analysis. Nat. Protoc..

[59] Roy A, Kucukural A, Zhang Y (2010). I-TASSER: A unified platform for automated protein structure and function prediction. Nat. Protoc..

[60] Zhang Y, Skolnick J (2005). TM-align: A protein structure alignment algorithm based on the TM-score. Nucleic Acids Res..

[61] Pettersen EF (2004). UCSF Chimera: A visualization system for exploratory research and analysis. J. Comput. Chem..

[62] Deng W (2016). Computational prediction of methylation types of covalently modified lysine and arginine residues in proteins. Brief. Bioinform..

[63] Pejaver V (2014). The structural and functional signatures of proteins that undergo multiple events of post-translational modification. Protein Sci..

[64] Blom N, Gammeltoft S, Brunak S (1999). Sequence and structure-based prediction of eukaryotic protein phosphorylation sites. J. Mol. Biol..

[65] Steentoft C (2013). Precision mapping of the human O-GalNAc glycoproteome through SimpleCell technology. Embo J..

[66] Pitti T (2019). N-GlyDE: a two-stage N-linked glycosylation site prediction incorporating gapped dipeptides and pattern-based encoding. Sci. Rep..

[67] Radivojac P (2010). Identification, analysis, and prediction of protein ubiquitination sites. Proteins.

[68] Li, A., Gao, X., Ren, J., Jin, C. & Xue, Y. BDM-PUB: Computational prediction of protein ubiquitination sites with a Bayesian discriminant method. *BDM-PUB: Computational Prediction of Protein Ubiquitination Sites with a Bayesian Discriminant Method* (2009).

[69] Warde-Farley D (2010). The GeneMANIA prediction server: Biological network integration for gene prioritization and predicting gene function. Nucleic Acids Res..

[70] Szklarczyk D (2019). STRING v11: protein-protein association networks with increased coverage, supporting functional discovery in genome-wide experimental datasets. Nucleic Acids Res..

