# Supplementary material for: Insilico prediction and functional analysis of nonsynonymous SNPs in human CTLA4 gene
Source: Sci Rep. 2022 Nov 28;12:20441. doi: 10.1038/s41598-022-24699-0 (PMC9705290; doi:10.1038/s41598-022-24699-0)
Supplement: Supplementary file 1 — Supplementary Information. [file 41598_2022_24699_MOESM1_ESM.zip › Supplementary Data/Figure S1.pdf]

ConSeq Results

```
1          11         21         31         41
MACLGFQRHK AQLNLAARTW PCTLLFFLLF IPVFCKAMHV AQPAVVLASS
eeeeebeeee bebeebeeeb ebbbbbbbbb bbbbbebbeb bbebbbbbee
f                                         s  sf    s

51         61         71         81         91
RGIASFVCEY ASPGKATEVR VTVLRQADSQ VTEVCAATYM MGNELTFLDD
eebbbbbbeb eeeeebeebe bebbeeeeee beebbbbebe eeeebebeee
 f s   s          f f  f  f         ss f

101        111        121        131        141
SICTGTSSGN QVNLTIQGLR AMDTGLYICK VELMYPPPYY LGIGNGTQIY
eebeeeeeee ebebbbebbe beeeebbbbe beebbebebe bbeeeebebb
  s f       sf s        f f s s   f   fsfs     f fs s

151        161        171        181        191
VIDPEPCPDS DFLLWILAAV SSGLFFYSFL LTAVSLSKML KKRSPLTTGV
bbeeeeeeeb ebbbbbbbbb bebbbbbbbb bbbbbbbebb eeeeeeeebb
s                            s                    ff

201        211        221
YVKMPPTEPE CEKQFQPYFI PIN
bbebeeeeee beeebeebeb eee
s f          f   f  s  ff
```

Legend:

The conservation scale:

1	2	3	4	5	6	7	8	9
Variable				Average				Conserved

e - An exposed residue according to the neural-network algorithm.

b - A buried residue according to the neural-network algorithm.

f - A predicted functional residue (highly conserved and exposed).

s - A predicted structural residue (highly conserved and buried).

X - Insufficient data - the calculation for this site was performed on less than 10% of the sequences.
