# Supplementary material for: Insilico prediction and functional analysis of nonsynonymous SNPs in human CTLA4 gene
Source: Sci Rep. 2022 Nov 28;12:20441. doi: 10.1038/s41598-022-24699-0 (PMC9705290; doi:10.1038/s41598-022-24699-0)
Supplement: Supplementary file 1 — Supplementary Information. [file 41598_2022_24699_MOESM1_ESM.zip › Supplementary Data/Table S1.docx]

**Table S1 :** Details of all nsSNPs obtained from dbSNP

| **S. NO** | **SNP IDs** | **Protein Accession Number** | **Positions** | **Substitutions** | **Global MAF** |
| --- | --- | --- | --- | --- | --- |
|  | [rs767352102](https://www.ncbi.nlm.nih.gov/SNP/snp_ref.cgi?rs=767352102) | AAL07473.1 | 2 | A [Ala]⇒S [Ser] | T=0./0 |
|  | [rs1289408071](https://www.ncbi.nlm.nih.gov/SNP/snp_ref.cgi?rs=1289408071) | AAL07473.1 | 3 | C [Cys]⇒ F [Phe] | T=0./0 |
|  | [rs896306346](https://www.ncbi.nlm.nih.gov/SNP/snp_ref.cgi?rs=896306346) | AAL07473.1 | 6 | F [Phe] ⇒ I [Ile] | A=0./0 |
|  | [rs201778935](https://www.ncbi.nlm.nih.gov/SNP/snp_ref.cgi?rs=201778935) | AAL07473.1 | 8 | R [Arg] ⇒ Q [Gln] | T=0.000023/2 |
|  | [rs138279736](https://www.ncbi.nlm.nih.gov/SNP/snp_ref.cgi?rs=138279736) | AAL07473.1 | 8 | R [Arg] ⇒ L [Leu] | A=0.000053/5 |
|  | [rs146541851](https://www.ncbi.nlm.nih.gov/SNP/snp_ref.cgi?rs=146541851) | AAL07473.1 | 10 | K [Lys]⇒ E [Glu] | G=0./0 |
|  | [rs1484954450](https://www.ncbi.nlm.nih.gov/SNP/snp_ref.cgi?rs=1484954450) | AAL07473.1 | 11 | A [Ala]⇒ G [Gly] | G=0.000004/1 |
|  | [rs748599835](https://www.ncbi.nlm.nih.gov/SNP/snp_ref.cgi?rs=748599835) | AAL07473.1 | 16 | A [Ala]⇒ T [Thr] | A=0.000004/1 |
|  | [rs772433747](https://www.ncbi.nlm.nih.gov/SNP/snp_ref.cgi?rs=772433747) | AAL07473.1 | 16 | A [Ala]⇒ D [Asp] | A=0.000032/8 |
|  | [rs1484109503](https://www.ncbi.nlm.nih.gov/SNP/snp_ref.cgi?rs=1484109503) | AAL07473.1 | 17 | T [Thr] ⇒ S [Ser] | G=0./0 |
|  | [rs1323841915](https://www.ncbi.nlm.nih.gov/SNP/snp_ref.cgi?rs=1323841915) | AAL07473.1 | 19 | T [Thr] ⇒ I [Ile] | T=0./0 |
|  | [rs769368847](https://www.ncbi.nlm.nih.gov/SNP/snp_ref.cgi?rs=769368847) | AAL07473.1 | 20 | W [Trp] ⇒ L [Leu] | T=0.000004/1 |
|  | [rs1041117695](https://www.ncbi.nlm.nih.gov/SNP/snp_ref.cgi?rs=1041117695) | AAL07473.1 | 21 | P [Pro] ⇒ R [Arg] | G=0.000012/3 |
|  | [rs1365829864](https://www.ncbi.nlm.nih.gov/SNP/snp_ref.cgi?rs=1365829864) | AAL07473.1 | 23 | T [Thr] ⇒ I [Ile] | T=0.000004/1 |
|  | [rs1280951195](https://www.ncbi.nlm.nih.gov/SNP/snp_ref.cgi?rs=1280951195) | AAL07473.1 | 25 | L [Leu] ⇒ P [Pro] | C=0./0 |
|  | [rs748802696](https://www.ncbi.nlm.nih.gov/SNP/snp_ref.cgi?rs=748802696) | AAL07473.1 | 26 | F [Phe] ⇒ S [Ser] | C=0.000004/1 |
|  | [rs774434261](https://www.ncbi.nlm.nih.gov/SNP/snp_ref.cgi?rs=774434261) | AAL07473.1 | 31 | I [Ile]⇒ V [Val] | G=0.000004/1 |
|  | [rs369567630](https://www.ncbi.nlm.nih.gov/SNP/snp_ref.cgi?rs=369567630) | AAL07473.1 | 32 | P [Pro] ⇒ S [Ser] | T=0./0 |
|  | [rs1413571467](https://www.ncbi.nlm.nih.gov/SNP/snp_ref.cgi?rs=1413571467) | AAL07473.1 | 32 | P [Pro] ⇒ L [Leu] | None |
|  | [rs1293454690](https://www.ncbi.nlm.nih.gov/SNP/snp_ref.cgi?rs=1293454690) | AAL07473.1 | 39 | H [His]⇒ P [Pro] | C=0./0 |
|  | [rs1553657378](https://www.ncbi.nlm.nih.gov/snp/rs1553657378) | AAL07473.1 | 40 | V [Val] ⇒ M [Met] | None |
|  | [rs766143912](https://www.ncbi.nlm.nih.gov/SNP/snp_ref.cgi?rs=766143912) | AAL07473.1 | 46 | V [Val] ⇒ I [Ile] | A=0.000004/1 |
|  | [rs759766975](https://www.ncbi.nlm.nih.gov/SNP/snp_ref.cgi?rs=759766975) | AAL07473.1 | 51 | R [Arg] ⇒ Q [Gln] | A=0.000004/1 |
|  | [rs1216841973](https://www.ncbi.nlm.nih.gov/SNP/snp_ref.cgi?rs=1216841973) | AAL07473.1 | 52 | G [Gly]⇒ S [Ser] | A=0./0 |
|  | [rs1553657387](https://www.ncbi.nlm.nih.gov/snp/rs1553657387) | AAL07473.1 | 54 | A [Ala]⇒ T [Thr] | None |
|  | [rs752811424](https://www.ncbi.nlm.nih.gov/SNP/snp_ref.cgi?rs=752811424) | AAL07473.1 | 60 | Y [Tyr] ⇒ F [Phe] | T=0.000004/1 |
|  | [rs1221748361](https://www.ncbi.nlm.nih.gov/SNP/snp_ref.cgi?rs=1221748361) | AAL07473.1 | 61 | A [Ala]⇒ T [Thr] | A=0.000004/1 |
|  | [rs758465752](https://www.ncbi.nlm.nih.gov/SNP/snp_ref.cgi?rs=758465752) | AAL07473.1 | 64 | G [Gly]⇒ D [Asp] | A=0.000004/1 |
|  | [rs1346241200](https://www.ncbi.nlm.nih.gov/SNP/snp_ref.cgi?rs=1346241200) | AAL07473.1 | 65 | K [Lys]⇒ E [Glu] | G=0.000004/1 |
|  | [rs557116456](https://www.ncbi.nlm.nih.gov/SNP/snp_ref.cgi?rs=557116456) | AAL07473.1 | 69 | V [Val] ⇒ I [Ile] | A=0./0 |
|  | [rs757566658](https://www.ncbi.nlm.nih.gov/SNP/snp_ref.cgi?rs=757566658) | AAL07473.1 | 69 | V [Val] ⇒ A [Ala] | C=0.000008/2 |
|  | [rs606231422](https://www.ncbi.nlm.nih.gov/snp/rs606231422) | AAL07473.1 | 70 | R [Arg] ⇒ W [Trp] | None |
|  | [rs1347670398](https://www.ncbi.nlm.nih.gov/SNP/snp_ref.cgi?rs=1347670398) | AAL07473.1 | 71 | V [Val] ⇒ L [Leu] | T=0./0 |
|  | [rs1206369281](https://www.ncbi.nlm.nih.gov/SNP/snp_ref.cgi?rs=1206369281) | AAL07473.1 | 72 | T [Thr] ⇒ A [Ala] | G=0.000004/1 |
|  | [rs781579729](https://www.ncbi.nlm.nih.gov/SNP/snp_ref.cgi?rs=781579729) | AAL07473.1 | 72 | T [Thr] ⇒ I [Ile] | T=0.000004/1 |
|  | [rs1196646336](https://www.ncbi.nlm.nih.gov/SNP/snp_ref.cgi?rs=1196646336) | AAL07473.1 | 75 | R [Arg] ⇒ L [Leu] | T=0.000008/2 |
|  | [rs1378866958](https://www.ncbi.nlm.nih.gov/SNP/snp_ref.cgi?rs=1378866958) | AAL07473.1 | 77 | A [Ala]⇒ T [Thr] | A=0.000004/1 |
|  | [rs754725143](https://www.ncbi.nlm.nih.gov/SNP/snp_ref.cgi?rs=754725143) | AAL07473.1 | 79 | S [Ser] ⇒ N [Asn] | A=0.000089/1 |
|  | [rs1431375309](https://www.ncbi.nlm.nih.gov/SNP/snp_ref.cgi?rs=1431375309) | AAL07473.1 | 81 | V [Val] ⇒ L [Leu] | C=0./0 |
|  | [rs1422926559](https://www.ncbi.nlm.nih.gov/SNP/snp_ref.cgi?rs=1422926559) | AAL07473.1 | 84 | V [Val] ⇒ I [Ile] | A=0./0 |
|  | [rs376038796](https://www.ncbi.nlm.nih.gov/SNP/snp_ref.cgi?rs=376038796) | AAL07473.1 | 86 | A [Ala]⇒ V [Val] | T=0.000089/1 |
|  | [rs1270679463](https://www.ncbi.nlm.nih.gov/SNP/snp_ref.cgi?rs=1270679463) | AAL07473.1 | 87 | A [Ala]⇒ T [Thr] | A=0./0 |
|  | [rs1403336336](https://www.ncbi.nlm.nih.gov/SNP/snp_ref.cgi?rs=1403336336) | AAL07473.1 | 89 | Y [Tyr] ⇒ H [His] | C=0.000004/1 |
|  | [rs1559591751](https://www.ncbi.nlm.nih.gov/snp/rs1559591751) | AAL07473.1 | 89 | Y [Tyr] ⇒ C [Cys] | None |
|  | [rs370443546](https://www.ncbi.nlm.nih.gov/SNP/snp_ref.cgi?rs=370443546) | AAL07473.1 | 90 | M [Met]⇒ V [Val] | G=0./0 |
|  | [rs746900785](https://www.ncbi.nlm.nih.gov/SNP/snp_ref.cgi?rs=746900785) | AAL07473.1 | 91 | M [Met]⇒ L [Leu] | T=0.000016/2 |
|  | [rs1065442](https://www.ncbi.nlm.nih.gov/SNP/snp_ref.cgi?rs=1065442) | AAL07473.1 | 91 | M [Met]⇒ T [Thr] | C=0./0 |
|  | [rs1052219132](https://www.ncbi.nlm.nih.gov/SNP/snp_ref.cgi?rs=1052219132) | AAL07473.1 | 91 | M [Met]⇒ I [Ile] | A=0./0 |
|  | [rs770666846](https://www.ncbi.nlm.nih.gov/SNP/snp_ref.cgi?rs=770666846) | AAL07473.1 | 94 | E [Glu] ⇒ V [Val] | T=0.000004/1 |
|  | [rs896360225](https://www.ncbi.nlm.nih.gov/SNP/snp_ref.cgi?rs=896360225) | AAL07473.1 | 102 | I [Ile]⇒ V [Val] | None |
|  | [rs776440178](https://www.ncbi.nlm.nih.gov/SNP/snp_ref.cgi?rs=776440178) | AAL07473.1 | 102 | I [Ile]⇒ M [Met] | G=0.000004/1 |
|  | [rs759232662](https://www.ncbi.nlm.nih.gov/SNP/snp_ref.cgi?rs=759232662) | AAL07473.1 | 104 | T [Thr] ⇒ M [Met] | T=0.000004/1 |
|  | [rs777843969](https://www.ncbi.nlm.nih.gov/SNP/snp_ref.cgi?rs=777843969) | AAL07473.1 | 106 | T [Thr] ⇒ N [Asn] | A=0.000004/1 |
|  | [rs144988077](https://www.ncbi.nlm.nih.gov/SNP/snp_ref.cgi?rs=144988077) | AAL07473.1 | 109 | G [Gly]⇒ E [Glu] | A=0.000508/45 |
|  | [rs763030646](https://www.ncbi.nlm.nih.gov/SNP/snp_ref.cgi?rs=763030646) | AAL07473.1 | 111 | Q [Gln] ⇒ L [Leu] | T=0.000004/1 |
|  | [rs1451326152](https://www.ncbi.nlm.nih.gov/SNP/snp_ref.cgi?rs=1451326152) | AAL07473.1 | 112 | V [Val] ⇒ M [Met] | A=0.000004/1 |
|  | [rs764089901](https://www.ncbi.nlm.nih.gov/SNP/snp_ref.cgi?rs=764089901) | AAL07473.1 | 118 | G [Gly]⇒ R [Arg] | C=0./0 |
|  | [rs752037577](https://www.ncbi.nlm.nih.gov/SNP/snp_ref.cgi?rs=752037577) | AAL07473.1 | 122 | M [Met]⇒ V [Val] | G=0./0 |
|  | [rs1422919298](https://www.ncbi.nlm.nih.gov/SNP/snp_ref.cgi?rs=1422919298) | AAL07473.1 | 122 | M [Met]⇒ T [Thr] | C=0.000004/1 |
|  | [rs1325759242](https://www.ncbi.nlm.nih.gov/SNP/snp_ref.cgi?rs=1325759242) | AAL07473.1 | 122 | M [Met]⇒ I [Ile] | A=0./0 |
|  | [rs1286462906](https://www.ncbi.nlm.nih.gov/SNP/snp_ref.cgi?rs=1286462906) | AAL07473.1 | 124 | T [Thr] ⇒ S [Ser] | None |
|  | [rs757773669](https://www.ncbi.nlm.nih.gov/SNP/snp_ref.cgi?rs=757773669) | AAL07473.1 | 124 | T [Thr] ⇒ M [Met] | T=0./0 |
|  | [rs1553657427](https://www.ncbi.nlm.nih.gov/snp/rs1553657427) | AAL07473.1 | 125 | G [Gly]⇒ R [Arg] | None |
|  | [rs1553657428](https://www.ncbi.nlm.nih.gov/snp/rs1553657428) | AAL07473.1 | 125 | G [Gly]⇒ E [Glu] | None |
|  | [rs750841862](https://www.ncbi.nlm.nih.gov/SNP/snp_ref.cgi?rs=750841862) | AAL07473.1 | 128 | I [Ile]⇒ M [Met] | G=0.000008/2 |
|  | [rs1559591813](https://www.ncbi.nlm.nih.gov/snp/rs1559591813) | AAL07473.1 | 131 | V [Tyr] ⇒ A [Ala] | None |
|  | [rs1294482526](https://www.ncbi.nlm.nih.gov/SNP/snp_ref.cgi?rs=1294482526) | AAL07473.1 | 134 | M [Met]⇒ V [Val] | None |
|  | [rs1553657429](https://www.ncbi.nlm.nih.gov/snp/rs1553657429) | AAL07473.1 | 137 | P [Pro] ⇒ L [Leu] | None |
|  | [rs1553657430](https://www.ncbi.nlm.nih.gov/snp/rs1553657430) | AAL07473.1 | 138 | P [Pro] ⇒ T [Thr] | None |
|  | [rs1357774202](https://www.ncbi.nlm.nih.gov/SNP/snp_ref.cgi?rs=1357774202) | AAL07473.1 | 141 | L [Leu] ⇒ Q [Gln] | A=0.000004/1 |
|  | [rs757989570](https://www.ncbi.nlm.nih.gov/SNP/snp_ref.cgi?rs=757989570) | AAL07473.1 | 143 | I [Ile]⇒ V [Val] | G=0.000004/1 |
|  | [rs1266819920](https://www.ncbi.nlm.nih.gov/SNP/snp_ref.cgi?rs=1266819920) | AAL07473.1 | 143 | I [Ile]⇒ T [Thr] | C=0.000004/1 |
|  | [rs1559591850](https://www.ncbi.nlm.nih.gov/snp/rs1559591850) | AAL07473.1 | 144 | G [Gly]⇒ C [Cys] | None |
|  | [rs1356678649](https://www.ncbi.nlm.nih.gov/SNP/snp_ref.cgi?rs=1356678649) | AAL07473.1 | 145 | N [Asn] ⇒ H [His] | C=0.000004/1 |
|  | [rs1204026047](https://www.ncbi.nlm.nih.gov/SNP/snp_ref.cgi?rs=1204026047) | AAL07473.1 | 145 | N [Asn] ⇒ S [Ser] | G=0.000004/1 |
|  | [rs1559591863](https://www.ncbi.nlm.nih.gov/snp/rs1559591863) | AAL07473.1 | 146 | G [Gly]⇒ L [Leu] | None |
|  | [rs1466152724](https://www.ncbi.nlm.nih.gov/SNP/snp_ref.cgi?rs=1466152724) | AAL07473.1 | 147 | T [Thr] ⇒ A [Ala] | G=0.000004/1 |
|  | [rs756706504](https://www.ncbi.nlm.nih.gov/SNP/snp_ref.cgi?rs=756706504) | AAL07473.1 | 156 | P [Pro] ⇒ L [Leu] | T=0.000008/1 |
|  | [rs745734610](https://www.ncbi.nlm.nih.gov/SNP/snp_ref.cgi?rs=745734610) | AAL07473.1 | 158 | P [Pro] ⇒ L [Leu] | T=0.000004/1 |
|  | [rs778733155](https://www.ncbi.nlm.nih.gov/SNP/snp_ref.cgi?rs=778733155) | AAL07473.1 | 160 | S [Ser]⇒ C [Cys] | G=0.002/1 |
|  | [rs1239916731](https://www.ncbi.nlm.nih.gov/snp/rs1239916731) | AAL07473.1 | 162 | R [Arg] ⇒ G [Gly] | G=0./0 |
|  | [rs749973402](https://www.ncbi.nlm.nih.gov/snp/rs749973402) | AAL07473.1 | 163 | G [Gly]⇒ C [Cys] | T=0.000004/1 |
|  | [rs1396254904](https://www.ncbi.nlm.nih.gov/SNP/snp_ref.cgi?rs=1396254904) | AAL07473.1 | 165 | W [Trp] ⇒ R [Arg] | C=0.000004/1 |
|  | [rs779775271](https://www.ncbi.nlm.nih.gov/SNP/snp_ref.cgi?rs=779775271) | AAL07473.1 | 166 | I [Ile]⇒ V [Val] | G=0.000004/1 |
|  | [rs74808460](https://www.ncbi.nlm.nih.gov/snp/rs74808460) | AAL07473.1 | 169 | P [Pro] ⇒ A [Ala] | G=0./0 |
|  | [rs1348195897](https://www.ncbi.nlm.nih.gov/SNP/snp_ref.cgi?rs=1348195897) | AAL07473.1 | 170 | V [Val] ⇒ F [Phe] | T=0./0 |
|  | [rs753717111](https://www.ncbi.nlm.nih.gov/snp/rs753717111) | AAL07473.1 | 171 | R [Arg] ⇒ G [Gly] | G=0./0 |
|  | [rs768961499](https://www.ncbi.nlm.nih.gov/SNP/snp_ref.cgi?rs=768961499) | AAL07473.1 | 171 | S [Ser] ⇒ T [Thr] | C=0.000004/1 |
|  | [rs963824682](https://www.ncbi.nlm.nih.gov/SNP/snp_ref.cgi?rs=963824682) | AAL07473.1 | 172 | S [Ser] ⇒ L [Leu] | T=0.000089/1 |
|  | [rs1310297644](https://www.ncbi.nlm.nih.gov/SNP/snp_ref.cgi?rs=1310297644) | AAL07473.1 | 180 | L [Leu] ⇒ F [Phe] | T=0.000004/1 |
|  | [rs771987351](https://www.ncbi.nlm.nih.gov/SNP/snp_ref.cgi?rs=771987351) | AAL07473.1 | 183 | A [Ala]⇒ S [Ser] | T=0.000004/1 |
|  | [rs773775010](https://www.ncbi.nlm.nih.gov/SNP/snp_ref.cgi?rs=773775010) | AAL07473.1 | 185 | S [Ser] ⇒ T [Thr] | A=0./0 |
|  | [rs761227535](https://www.ncbi.nlm.nih.gov/SNP/snp_ref.cgi?rs=761227535) | AAL07473.1 | 186 | L [Leu] ⇒ S [Ser] | C=0./0 |
|  | [rs1202814386](https://www.ncbi.nlm.nih.gov/SNP/snp_ref.cgi?rs=1202814386) | AAL07473.1 | 187 | S [Ser] ⇒ R [Arg] | C=0.000004/1 |
|  | [rs766875859](https://www.ncbi.nlm.nih.gov/SNP/snp_ref.cgi?rs=766875859) | AAL07473.1 | 188 | K [Lys]⇒ R[Arg] | G=0./0 |
|  | [rs199912925](https://www.ncbi.nlm.nih.gov/SNP/snp_ref.cgi?rs=199912925) | AAL07473.1 | 189 | M [Met]⇒ V [Val] | G=0./0 |
|  | [rs745310078](https://www.ncbi.nlm.nih.gov/snp/rs745310078) | AAL07473.1 | 194 | S [Ser] ⇒ R [Arg] | T=0.000004/1 |
|  | [rs1440286766](https://www.ncbi.nlm.nih.gov/SNP/snp_ref.cgi?rs=1440286766) | AAL07473.1 | 194 | S [Ser] ⇒ N [Asn] | A=0./0 |
|  | [rs1440097102](https://www.ncbi.nlm.nih.gov/SNP/snp_ref.cgi?rs=1440097102) | AAL07473.1 | 195 | P [Pro] ⇒ R [Arg] | G=0.00003/1 |
|  | [rs767174634](https://www.ncbi.nlm.nih.gov/SNP/snp_ref.cgi?rs=767174634) | AAL07473.1 | 198 | T [Thr] ⇒ K [Lys] | A=0.000008/1 |
|  | [rs1361102216](https://www.ncbi.nlm.nih.gov/SNP/snp_ref.cgi?rs=1361102216) | AAL07473.1 | 199 | G [Gly]⇒ R [Arg] | A=0.000004/1 |
|  | [rs1046533169](https://www.ncbi.nlm.nih.gov/SNP/snp_ref.cgi?rs=1046533169) | AAL07473.1 | 200 | V [Val] ⇒ F [Phe] | None |
|  | [rs755615887](https://www.ncbi.nlm.nih.gov/SNP/snp_ref.cgi?rs=755615887) | AAL07473.1 | 200 | V [Val] ⇒ A [Ala] | C=0./0 |
|  | [rs1205829960](https://www.ncbi.nlm.nih.gov/SNP/snp_ref.cgi?rs=1205829960) | AAL07473.1 | 205 | P [Pro] ⇒ A [Ala] | G=0.00003/1 |
|  | [rs1245023275](https://www.ncbi.nlm.nih.gov/SNP/snp_ref.cgi?rs=1245023275) | AAL07473.1 | 206 | P [Pro] ⇒ A [Ala] | G=0.000004/1 |
|  | [rs1278638729](https://www.ncbi.nlm.nih.gov/SNP/snp_ref.cgi?rs=1278638729) | AAL07473.1 | 207 | T [Thr] ⇒ A [Ala] | G=0./0 |
|  | [rs754809262](https://www.ncbi.nlm.nih.gov/SNP/snp_ref.cgi?rs=754809262) | AAL07473.1 | 208 | E [Glu] ⇒ D [Asp] | A=0./0 |
|  | [rs778534474](https://www.ncbi.nlm.nih.gov/SNP/snp_ref.cgi?rs=778534474) | AAL07473.1 | 209 | P [Pro] ⇒ R [Arg] | G=0.000111/1 |
|  | [rs527697475](https://www.ncbi.nlm.nih.gov/SNP/snp_ref.cgi?rs=527697475) | AAL07473.1 | 211 | C [Cys]⇒ R [Arg] | C=0./0 |
|  | [rs367760388](https://www.ncbi.nlm.nih.gov/SNP/snp_ref.cgi?rs=367760388) | AAL07473.1 | 222 | I [Ile]⇒ V [Val] | None |
|  | [rs1444367175](https://www.ncbi.nlm.nih.gov/SNP/snp_ref.cgi?rs=1444367175) | AAL07473.1 | 223 | N [Asn] ⇒ S [Ser] | G=0.000004/1 |
