# Supplementary material for: Insilico prediction and functional analysis of nonsynonymous SNPs in human CTLA4 gene
Source: Sci Rep. 2022 Nov 28;12:20441. doi: 10.1038/s41598-022-24699-0 (PMC9705290; doi:10.1038/s41598-022-24699-0)
Supplement: Supplementary file 1 — Supplementary Information. [file 41598_2022_24699_MOESM1_ESM.zip › Supplementary Data/Table S2 .docx]

**Table S2:** The results for all the nsSNPs located in CTLA4 gene by SIFT, PROVEAN, and PhD-SNP

|  |  | **SIFT** | | **PROVEAN** | | **PhD-SNP** | | |
| --- | --- | --- | --- | --- | --- | --- | --- | --- |
| **Residual Change** | **Position** | **Prediction** | **Tolerance index** | **Score** | **Prediction (cutoff= -2.5)** | **Prediction** | **RI** | **Probability** |
| A [Ala]⇒S [Ser] | 2 | AFFECTED | 0.00 | -0.520 | Neutral | Neutral | 7 | 0.125 |
| C [Cys]⇒ F [Phe] | 3 | TOLERATED | 0.22 | -1.116 | Neutral | Neutral | 5 | 0.238 |
| F [Phe] ⇒ I [Ile] | 6 | TOLERATED | 0.12 | -0.731 | Neutral | Neutral | 5 | 0.249 |
| R [Arg] ⇒ Q [Gln] | 8 |  |  | -0.815 | Neutral | Disease | 0 | 0.525 |
| R [Arg] ⇒ L [Leu] | 8 | TOLERATED | 0.05 | -1.496 | Neutral | Disease | 3 | 0.639 |
| K [Lys]⇒ E [Glu] | 10 | TOLERATED | 0.31 | -0.338 | Neutral | Neutral | 5 | 0.259 |
| A [Ala]⇒ G [Gly] | 11 | TOLERATED | 0.06 | -0.634 | Neutral | Neutral | 4 | 0.283 |
| A [Ala]⇒ T [Thr] | 16 |  |  | -0.258 | Neutral | Neutral | 3 | 0.337 |
| A [Ala]⇒ D [Asp] | 16 | TOLERATED | 0.15 | -0.759 | Neutral | Disease | 3 | 0.632 |
| T [Thr] ⇒ S [Ser] | 17 |  |  |  |  |  |  |  |
| T [Thr] ⇒ I [Ile] | 19 | AFFECTED | 0.00 | -1.393 | Neutral | Disease | 3 | 0.665 |
| W [Trp] ⇒ L [Leu] | 20 | AFFECTED | 0.00 | -2.615 | Deleterious | Disease | 6 | 0.803 |
| P [Pro] ⇒ R [Arg] | 21 | AFFECTED | 0.02 | -1.974 | Neutral | Disease | 4 | 0.701 |
| T [Thr] ⇒ I [Ile] | 23 | TOLERATED | 0.23 | -0.975 | Neutral | Disease | 4 | 0.694 |
| L [Leu] ⇒ P [Pro] | 25 | AFFECTED | 0.00 | -2.233 | Neutral | Disease | 8 | 0.883 |
| F [Phe] ⇒ S [Ser] | 26 | AFFECTED | 0.03 | -1.237 | Neutral | Disease | 3 | 0.659 |
| I [Ile]⇒ V [Val] | 31 | TOLERATED | 0.15 | -0.266 | Neutral | Neutral | 6 | 0.221 |
| P [Pro] ⇒ S [Ser] | 32 |  |  | -2.039 | Neutral | Disease | 2 | 0.578 |
| P [Pro] ⇒ L [Leu] | 32 | AFFECTED | 0.01 | -2.072 | Neutral | Disease | 5 | 0.746 |
| H [His]⇒ P [Pro] | 39 | TOLERATED | 0.25 | -1.893 | Neutral | Disease | 4 | 0.711 |
| V [Val] ⇒ M [Met] | 40 | AFFECTED | 0.01 | -1.716 | Neutral | Disease | 2 | 0.592 |
| V [Val] ⇒ I [Ile] | 46 | TOLERATED | 0.20 | -0.063 | Neutral | Neutral | 7 | 0.174 |
| R [Arg] ⇒ Q [Gln] | 51 | TOLERATED | 0.17 | -0.431 | Neutral | Neutral | 5 | 0.232 |
| G [Gly]⇒ S [Ser] | 52 | TOLERATED | 0.19 | -1.373 | Neutral | Disease | 2 | 0.602 |
| A [Ala]⇒ T [Thr] | 54 | TOLERATED | 0.08 | -1.262 | Neutral | Disease | 1 | 0.544 |
| Y [Tyr] ⇒ F [Phe] | 60 | AFFECTED | 0.00 | -2.125 | Neutral | Disease | 2 | 0.619 |
| A [Ala]⇒ T [Thr] | 61 | TOLERATED | 0.91 | 0.794 | Neutral | Neutral | 9 | 0.037 |
| G [Gly]⇒ D [Asp] | 64 | TOLERATED | 0.23 | -1.171 | Neutral | Neutral | 0 | 0.481 |
| K [Lys]⇒ E [Glu] | 65 | TOLERATED | 0.65 | -0.592 | Neutral | Neutral | 1 | 0.451 |
| V [Val] ⇒ I [Ile] | 69 |  |  | 0.196 | Neutral | Neutral | 9 | 0.059 |
| V [Val] ⇒ A [Ala] | 69 | TOLERATED | 0.15 | -1.342 | Neutral | Neutral | 5 | 0.259 |
| R [Arg] ⇒ W [Trp] | 70 | AFFECTED | 0.00 | -4.816 | Deleterious | Disease | 3 | 0.627 |
| V [Val] ⇒ L [Leu] | 71 | TOLERATED | 0.15 | -1.330 | Neutral | Neutral | 6 | 0.176 |
| T [Thr] ⇒ A [Ala] | 72 |  |  | -1.478 | Neutral | Neutral | 5 | 0.263 |
| T [Thr] ⇒ I [Ile] | 72 | AFFECTED | 0.01 | -3.464 | Deleterious | Neutral | 3 | 0.355 |
| R [Arg] ⇒ L [Leu] | 75 | AFFECTED | 0.02 | -4.122 | Deleterious | Disease | 1 | 0.561 |
| A [Ala]⇒ T [Thr] | 77 | TOLERATED | 0.51 | 0.336 | Neutral | Neutral | 9 | 0.034 |
| S [Ser] ⇒ N [Asn] | 79 | TOLERATED | 0.07 | -0.263 | Neutral | Neutral | 5 | 0.247 |
| V [Val] ⇒ L [Leu] | 81 | TOLERATED | 0.17 | -0.966 | Neutral | Neutral | 4 | 0.312 |
| V [Val] ⇒ I [Ile] | 84 | AFFECTED | 0.00 | -0.512 | Neutral | Neutral | 5 | 0.229 |
| A [Ala]⇒ V [Val] | 86 | TOLERATED | 1.00 | -0.895 | Neutral | Neutral | 8 | 0.125 |
| A [Ala]⇒ T [Thr] | 87 | TOLERATED | 0.48 | 0.036 | Neutral | Neutral | 9 | 0.044 |
| Y [Tyr] ⇒ H [His] | 89 |  |  | -2.429 | Neutral | Neutral | 2 | 0.415 |
| Y [Tyr] ⇒ C [Cys] | 89 | TOLERATED | 0.10 | -3.790 | Deleterious | Disease | 0 | 0.521 |
| M [Met]⇒ V [Val] | 90 | TOLERATED | 0.14 | -0.160 | Neutral | Neutral | 7 | 0.155 |
| M [Met]⇒ L [Leu] | 91 |  |  | -0.497 | Neutral | Neutral | 7 | 0.162 |
| M [Met]⇒ T [Thr] | 91 |  |  | 0.563 | Neutral | Neutral | 8 | 0.089 |
| M [Met]⇒ I [Ile] | 91 | TOLERATED | 0.39 | -0.193 | Neutral | Neutral | 7 | 0.122 |
| E [Glu] ⇒ V [Val] | 94 | TOLERATED | 0.23 | -1.944 | Neutral | Neutral | 5 | 0.274 |
| I [Ile]⇒ V [Val] | 102 |  |  | -0.003 | Neutral | Neutral | 9 | 0.036 |
| I [Ile]⇒ M [Met] | 102 | TOLERATED | 0.25 | 0.381 | Neutral | Neutral | 8 | 0.075 |
| T [Thr] ⇒ M [Met] | 104 | TOLERATED | 0.07 | -1.188 | Neutral | Neutral | 7 | 0.130 |
| T [Thr] ⇒ N [Asn] | 106 | TOLERATED | 0.54 | -0.119 | Neutral | Neutral | 6 | 0.191 |
| G [Gly]⇒ E [Glu] | 109 | TOLERATED | 0.70 | 0.069 | Neutral | Neutral | 7 | 0.142 |
| Q [Gln] ⇒ L [Leu] | 111 | TOLERATED | 0.14 | -2.095 | Neutral | Neutral | 7 | 0.147 |
| V [Val] ⇒ M [Met] | 112 | AFFECTED | 0.00 | -2.008 | Neutral | Neutral | 0 | 0.494 |
| G [Gly]⇒ R [Arg] | 118 | AFFECTED | 0.04 | -3.635 | Deleterious | Disease | 4 | 0.721 |
| M [Met]⇒ V [Val] | 122 |  |  | -0.733 | Neutral | Neutral | 7 | 0.174 |
| M [Met]⇒ T [Thr] | 122 |  |  | 0.622 | Neutral | Neutral | 6 | 0.217 |
| M [Met]⇒ I [Ile] | 122 | TOLERATED | 0.42 | -0.885 | Neutral | Neutral | 6 | 0.206 |
| T [Thr] ⇒ S [Ser] | 124 |  |  | -2.937 | Deleterious | Disease | 3 | 0.670 |
| T [Thr] ⇒ M [Met] | 124 | AFFECTED | 0.00 | -4.417 | Deleterious | Disease | 5 | 0.748 |
| G [Gly]⇒ R [Arg] | 125 |  |  | -3.531 | Deleterious | Disease | 4 | 0.704 |
| G [Gly]⇒ E [Glu] | 125 | TOLERATED | 0.31 | -1.520 | Neutral | Disease | 3 | 0.674 |
| I [Ile]⇒ M [Met] | 128 | TOLERATED | 0.19 | -0.731 | Neutral | Neutral | 4 | 0.309 |
| V [Tyr] ⇒ A [Ala] | 131 | AFFECTED | 0.01 | -3.400 | Deleterious | Neutral | 4 | 0.324 |
| M [Met]⇒ V [Val] | 134 | AFFECTED | 0.03 | -3.044 | Deleterious | Disease | 5 | 0.763 |
| P [Pro] ⇒ L [Leu] | 137 | AFFECTED | 0.00 | -8.336 | Deleterious | Disease | 7 | 0.846 |
| P [Pro] ⇒ T [Thr] | 138 | AFFECTED | 0.00 | -6.776 | Deleterious | Disease | 5 | 0.766 |
| L [Leu] ⇒ Q [Gln] | 141 | TOLERATED | 0.48 | 0.240 | Neutral | Neutral | 5 | 0.225 |
| I [Ile]⇒ V [Val] | 143 |  |  | 0.287 | Neutral | Neutral | 4 | 0.284 |
| I [Ile]⇒ T [Thr] | 143 | TOLERATED | 0.20 | -0.161 | Neutral | Neutral | 0 | 0.485 |
| G [Gly]⇒ C [Cys] | 144 | TOLERATED | 0.10 | -4.424 | Deleterious | Disease | 6 | 0.784 |
| N [Asn] ⇒ H [His] | 145 |  |  | -4.185 | Deleterious | Disease | 5 | 0.747 |
| N [Asn] ⇒ S [Ser] | 145 | AFFECTED | 0.00 | -4.010 | Deleterious | Disease | 4 | 0.707 |
| G [Gly]⇒ L [Leu] | 146 | AFFECTED | 0.00 | -8.669 | Deleterious | Disease | 8 | 0.908 |
| T [Thr] ⇒ A [Ala] | 147 | AFFECTED | 0.00 | -4.638 | Deleterious | Disease | 3 | 0.628 |
| P [Pro] ⇒ L [Leu] | 156 | TOLERATED | 1.00 | -1.215 | Neutral | Neutral | 3 | 0.333 |
| P [Pro] ⇒ L [Leu] | 158 | AFFECTED | 0.02 | -4.111 | Deleterious | Disease | 7 | 0.866 |
| S [Ser]⇒ C [Cys] | 160 | AFFECTED | 0.01 | -1.970 | Neutral | Disease | 1 | 0.529 |
| R [Arg] ⇒ G [Gly] | 162 |  |  |  |  |  |  |  |
| G [Gly]⇒ C [Cys] | 163 |  |  |  |  |  |  |  |
| W [Trp] ⇒ R [Arg] | 165 | AFFECTED | 0.00 | -6.824 | Deleterious | Disease | 6 | 0.808 |
| I [Ile]⇒ V [Val] | 166 | TOLERATED | 1.00 | 0.063 | Neutral | Neutral | 8 | 0.084 |
| P [Pro] ⇒ A [Ala] | 169 |  |  |  |  |  |  |  |
| V [Val] ⇒ F [Phe] | 170 | AFFECTED | 0.01 | -2.551 | Deleterious | Disease | 6 | 0.802 |
| R [Arg] ⇒ G [Gly] | 171 |  |  |  |  |  |  |  |
| S [Ser] ⇒ T [Thr] | 171 | TOLERATED | 0.53 | -0.336 | Neutral | Neutral | 5 | 0.269 |
| S [Ser] ⇒ L [Leu] | 172 | TOLERATED | 0.22 | -1.684 | Neutral | Disease | 4 | 0.693 |
| L [Leu] ⇒ F [Phe] | 180 | AFFECTED | 0.00 | -1.964 | Neutral | Neutral | 2 | 0.411 |
| A [Ala]⇒ S [Ser] | 183 | AFFECTED | 0.03 | -1.699 | Neutral | Neutral | 1 | 0.466 |
| S [Ser] ⇒ T [Thr] | 185 | TOLERATED | 0.64 | -0.420 | Neutral | Neutral | 7 | 0.153 |
| L [Leu] ⇒ S [Ser] | 186 | TOLERATED | 0.08 | -1.691 | Neutral | Neutral | 4 | 0.277 |
| S [Ser] ⇒ R [Arg] | 187 | TOLERATED | 0.33 | -1.359 | Neutral | Neutral | 5 | 0.238 |
| K [Lys]⇒ R[Arg] | 188 | TOLERATED | 0.84 | -0.099 | Neutral | Neutral | 8 | 0.088 |
| M [Met]⇒ V [Val] | 189 | TOLERATED | 0.46 | -1.207 | Neutral | Neutral | 8 | 0.122 |
| S [Ser] ⇒ R [Arg] | 194 |  |  | -1.016 | Neutral | Neutral | 2 | 0.398 |
| S [Ser] ⇒ N [Asn] | 194 | TOLERATED | 0.76 | 0.329 | Neutral | Neutral | 8 | 0.118 |
| P [Pro] ⇒ R [Arg] | 195 | TOLERATED | 0.88 | 2.451 | Neutral | Neutral | 8 | 0.093 |
| T [Thr] ⇒ K [Lys] | 198 | TOLERATED | 0.86 | -0.267 | Neutral | Neutral | 1 | 0.468 |
| G [Gly]⇒ R [Arg] | 199 | TOLERATED | 0.09 | -2.142 | Neutral | Neutral | 1 | 0.440 |
| V [Val] ⇒ F [Phe] | 200 |  |  | -1.076 | Neutral | Neutral | 3 | 0.351 |
| V [Val] ⇒ A [Ala] | 200 | TOLERATED | 0.67 | -0.335 | Neutral | Neutral | 6 | 0.197 |
| P [Pro] ⇒ A [Ala] | 205 | TOLERATED | 0.47 | -0.579 | Neutral | Neutral | 7 | 0.136 |
| P [Pro] ⇒ A [Ala] | 206 | AFFECTED | 0.00 | -3.624 | Deleterious | Neutral | 2 | 0.381 |
| T [Thr] ⇒ A [Ala] | 207 | TOLERATED | 0.28 | -0.764 | Neutral | Neutral | 7 | 0.146 |
| E [Glu] ⇒ D [Asp] | 208 | TOLERATED | 0.29 | -0.927 | Neutral | Neutral | 6 | 0.180 |
| P [Pro] ⇒ R [Arg] | 209 | AFFECTED | 0.02 | -3.594 | Deleterious | Disease | 1 | 0.537 |
| C [Cys]⇒ R [Arg] | 211 | TOLERATED | 0.11 | -1.503 | Neutral | Neutral | 1 | 0.460 |
| I [Ile]⇒ V [Val] | 222 | AFFECTED | 0.00 | 0.273 | Neutral | Neutral | 9 | 0.026 |
| N [Asn] ⇒ S [Ser] | 223 | AFFECTED | 0.00 | -1.406 | Neutral | Neutral | 8 | 0.098 |
