# Supplementary material for: Insilico prediction and functional analysis of nonsynonymous SNPs in human CTLA4 gene
Source: Sci Rep. 2022 Nov 28;12:20441. doi: 10.1038/s41598-022-24699-0 (PMC9705290; doi:10.1038/s41598-022-24699-0)
Supplement: Supplementary file 1 — Supplementary Information. [file 41598_2022_24699_MOESM1_ESM.zip › Supplementary Data/Table S3.docx]

**Table S3:** Polyphen2 results for 28 selected nsSNPs from other tools

|  | R8L | Probably Damaging | 0.985 |
| --- | --- | --- | --- |
|  | R8Q | Probably Damaging | 0.993 |
|  | T19I | Possibly Damaging | 0.716 |
|  | P21R | Possibly Damaging | 0.843 |
|  | L25P | Probably Damaging | 0.991 |
|  | G52S | Probably Damaging | 0.992 |
|  | Y60F | Possibly Damaging | 0.938 |
|  | R70W | Probably Damaging | 1.000 |
|  | T72I | Probably Damaging | 0.995 |
|  | R75L | Probably Damaging | 0.983 |
|  | A86V | Probably Damaging | 1.000 |
|  | V112M | Probably Damaging | 1.000 |
|  | G118R | Probably Damaging | 1.000 |
|  | M134V | Possibly Damaging | 0.495 |
|  | P137L | Probably Damaging | 1.000 |
|  | P138T | Probably Damaging | 1.000 |
|  | N145H | Probably Damaging | 1.000 |
|  | N145S | Probably Damaging | 0.999 |
|  | G146L | Probably Damaging | 1.000 |
|  | T147A | Probably Damaging | 1.000 |
|  | P158L | Probably Damaging | 0.973 |
|  | S160C | Probably Damaging | 0.991 |
|  | W165R | Possibly Damaging | 0.838 |
|  | V170F | Possibly Damaging | 0.716 |
|  | L180F | Possibly Damaging | 0.838 |
|  | G199R | Probably Damaging | 1.000 |
|  | P209R | Probably Damaging | 1.000 |
|  | C211R | Possibly Damaging | 0.893 |
