# Supplementary material for: Insilico prediction and functional analysis of nonsynonymous SNPs in human CTLA4 gene
Source: Sci Rep. 2022 Nov 28;12:20441. doi: 10.1038/s41598-022-24699-0 (PMC9705290; doi:10.1038/s41598-022-24699-0)
Supplement: Supplementary file 1 — Supplementary Information. [file 41598_2022_24699_MOESM1_ESM.zip › Supplementary Data/Table S4.docx]

**Table S4:** Effects of nsSNPs on structural & functional properties of CTLA4 by MutPred2 server.

| **Substitution** | **Probability of deleterious substitution** | **Top Features** | **Affected PROSITE and ELM Motifs** |
| --- | --- | --- | --- |
| R70W | 0.634 | Loss of Helix  (P= 9.4e-03) | ELME000062 |
|  |  | Altered Transmembrane protein  (P= 4.0e-04) |  |
|  |  | Gain of Strand  (P= 0.03) |  |
| G118R | 0.778 | Altered Transmembrane protein  (P=8.7e-04) | ELME000193 |
|  |  | Altered Ordered interface  (P=0.05) |  |
|  |  | Gain of Relative solvent accessibility  (P=0.04) |  |
|  |  | Gain of N-linked glycosylation at N113  (P=2.1e-03) |  |
| P137L | 0.810 | Altered Ordered interface  (P=5.2e-03) | ELME000003 |
|  |  | Altered Transmembrane protein  (P= 7.2e-04) |  |
|  |  | Gain of Sulfation at Y135  (P=0.02) |  |
| P138T | 0.800 | Altered Ordered interface  (P= 0.01) | ELME000003 ELME000052  ELME000142 |
|  |  | Altered Transmembrane protein  (P= 1.1e-03) |  |
|  |  | Gain of Sulfation at Y135  (P= 0.02) |  |
| N145S | 0.179 | None | None |
| G146L | 0.854 | Altered Transmembrane protein  (P=7.4e-04) | ELME000070 ELME000202  PS00001  PS00008 |
|  |  | Loss of N-linked glycosylation at N145  (P= 6.1e-03) |  |
|  |  | Loss of Sulfation at Y150  (P= 0.03) |  |
| T147A | 0.426 | None | None |
| P209R | 0.533 | Altered Disordered interface  (P= 0.01) | ELME000005 ELME000064 ELME000220 PS00005  PS00006 |
|  |  | Loss of B-factor  (P= 0.03) |  |
|  |  | Altered Transmembrane protein  (P=0.04) |  |
