# Supplementary material for: Insilico prediction and functional analysis of nonsynonymous SNPs in human CTLA4 gene
Source: Sci Rep. 2022 Nov 28;12:20441. doi: 10.1038/s41598-022-24699-0 (PMC9705290; doi:10.1038/s41598-022-24699-0)

**Table S5:** Prediction of Phosphorylation Sites by NetPhos 3.1 and ModPred

|  | **NetPhos 3.1** | | | **ModPred** | | |
| --- | --- | --- | --- | --- | --- | --- |
|  | **Position** | **Score** | **Kinase** | **Position** | **Score** | **Confidence** |
| Serine (S) | 49 | 0.671 | PKC | 62* | 0.57 | Low |
|  | 62* | 0.652 | Unsp | 194* | 0.72 | Medium |
|  | 101 | 0.511 | PKA |  |  |  |
|  | 107 | 0.864 | Unsp |  |  |  |
|  | 171 | 0.526 | Cdc2 |  |  |  |
|  | 172 | 0.541 | Cdc2 |  |  |  |
|  | 178 | 0.563 | Cdc2 |  |  |  |
|  | 187 | 0.694 | PKC |  |  |  |
|  | 194* | 0.922 | unsp |  |  |  |
|  |  | | | | | |
| Threonine (T) | 67 | 0.645 | Unps | 197* | 0.53 | Low |
|  | 96 | 0.512 | CKI | 207* | 0.58 | Low |
|  | 104 | 0.618 | PKC |  |  |  |
|  | 106 | 0.693 | PKC |  |  |  |
|  | 147 | 0.591 | DNAPK |  |  |  |
|  | 197* | 0.718 | PKC |  |  |  |
|  | 198 | 0.624 | PKC |  |  |  |
|  | 207* | 0.610 | CKIT |  |  |  |
|  |  | | | | | |
| Tyrosine (Y) | 60* | 0.807 | Unsp | 60* | 0.55 | Low |
|  | 140 | 0.652 | Unps | 201* | 0.61 | Low |
|  | 201* | 0.835 | Unsp | 218 | 0.51 | Low |
|  |  | | | | | |

*Common in both NetPhos 3.1 and ModPred.


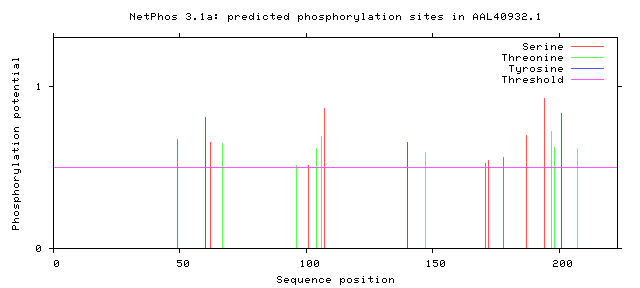

Supplement: Supplementary file 1 — Supplementary Information. [file 41598_2022_24699_MOESM1_ESM.zip › Supplementary Data/Table S5.docx]
