# Supplementary material for: Insilico prediction and functional analysis of nonsynonymous SNPs in human CTLA4 gene
Source: Sci Rep. 2022 Nov 28;12:20441. doi: 10.1038/s41598-022-24699-0 (PMC9705290; doi:10.1038/s41598-022-24699-0)
Supplement: Supplementary file 1 — Supplementary Information. [file 41598_2022_24699_MOESM1_ESM.zip › Supplementary Data/Table S6.docx]

**Table S6:** NetOGlyc 4.0 Results for CTLA4 (Wild type and Mutants)

| **Position** | **Score** | | | | | | | | |
| --- | --- | --- | --- | --- | --- | --- | --- | --- | --- |
|  | **Wild type** | **R70W** | **G118R** | **P137L** | **P138T** | **N145S** | **G146L** | **T147A** | **P209R** |
| 19 | 0.00625798 | 0.00631726 | 0.00644904 | 0.0201065 | 0.00596203 | 0.0049623 | 0.00590931 | 0.0056943 | 0.0064705 |
| 23 | 0.0184789 | 0.0194891 | 0.0204653 | 0.020381 | 0.0207207 | 0.0155942 | 0.0201154 | 0.0194704 | 0.0192636 |
| 49 | 0.114611 | 0.11548 | 0.11867 | 0.0166039 | 0.13606 | 0.114226 | 0.122208 | 0.133053 | 0.12867 |
| 50 | 0.0641703 | 0.067463 | 0.0671433 | 0.0085353 | 0.0753041 | 0.0590994 | 0.0641945 | 0.0740453 | 0.0707884 |
| 55 | 0.0183781 | 0.0179163 | 0.0182442 | 1.15922e-05 | 0.0175825 | 0.0169149 | 0.0176969 | 0.0181071 | 0.0189245 |
| 62 | 0.0488888 | 0.0459051 | 0.0440352 | 0.00456629 | 0.0407534 | 0.0432908 | 0.0412657 | 0.0412179 | 0.0443054 |
| 67 | 0.0256874 | 0.0147131 | 0.0240838 | 0.00282218 | 0.0264917 | 0.0236922 | 0.0250664 | 0.0254875 | 0.0239467 |
| 72 | 0.0753617 | 0.0426978 | 0.0719774 | 0.00806218 | 0.0713565 | 0.0718073 | 0.0693743 | 0.0713947 | 0.0792497 |
| 79 | 0.192383 | 0.12502 | 0.202324 | 0.0243413 | 0.19383 | 0.201392 | 0.188515 | 0.190927 | 0.212342 |
| 82 | 0.112855 | 0.112415 | 0.117165 | 0.0125488 | 0.107609 | 0.112855 | 0.113274 | 0.110663 | 0.123949 |
| 88 | 0.0589707 | 0.0600699 | 0.0625628 | 0.0074618 | 0.0686123 | 0.0593414 | 0.061521 | 0.0659989 | 0.0650981 |
| 96 | 0.0365513 | 0.0378642 | 0.0386629 | 0.00480094 | 0.0451375 | 0.039706 | 0.0426313 | 0.0443377 | 0.043515 |
| 101 | 0.0135063 | 0.0145421 | 0.0142153 | 8.19911e-06 | 0.0162296 | 0.0128751 | 0.0161092 | 0.015654 | 0.0141411 |
| 104 | 0.0296944 | 0.0328304 | 0.0324338 | 0.00396207 | 0.0392216 | 0.0281895 | 0.0332605 | 0.0363227 | 0.0319639 |
| 106 | 0.0673653 | 0.0681199 | 0.0670456 | 0.00932147 | 0.0834605 | 0.057135 | 0.0754891 | 0.0811741 | 0.0677901 |
| 107 | 0.0993579 | 0.103958 | 0.102207 | 0.0134941 | 0.121813 | 0.0864703 | 0.110914 | 0.114996 | 0.101746 |
| 108 | 0.0783428 | 0.0872864 | 0.106456 | 0.0113626 | 0.104178 | 0.0715314 | 0.0917356 | 0.0993134 | 0.0887444 |
| 115 | 0.0283135 | 0.0316083 | 0.0328673 | 0.00376251 | 0.0360843 | 0.0235368 | 0.0292612 | 0.0334777 | 0.0300831 |
| 124 | 0.0068308 | 0.00710709 | 0.00909042 | 1.75127e-06 | 0.00740228 | 0.00601372 | 0.00676273 | 0.00705562 | 0.00716501 |
| 138 |  |  |  |  | 0.00487903 |  |  |  |  |
| 145 |  |  |  |  |  | 0.0165973 |  |  |  |
| 147 | 0.0544976 | 0.0512284 | 0.0558789 | 0.0158481 | 0.0518585 | 0.0611041 | 0.0379547 |  | 0.0583383 |
| 160 | 0.0054857 | 0.00513075 | 0.00491289 | 0.00498604 | 0.00492885 | 0.00480827 | 0.00507206 | 0.00498825 | 0.00494921 |
| 171 | 0.0219476 | 0.0199406 | 0.0197907 | 0.0206128 | 0.0207311 | 0.0215568 | 0.0204584 | 0.0197054 | 0.0205173 |
| 172 | 0.00794835 | 0.00734223 | 0.00732452 | 0.00854266 | 0.0082924 | 0.00808738 | 0.00848516 | 0.00822879 | 0.00811185 |
| 178 | 0.0253519 | 0.023366 | 0.0239025 | 0.0240714 | 0.0235385 | 0.0259206 | 0.023978 | 0.0236551 | 0.0237563 |
| 182 | 0.00575182 | 0.00549861 | 0.00552958 | 0.0056069 | 0.00560563 | 0.00592486 | 0.00558251 | 0.00546128 | 0.00571123 |
| 185 | 0.0183938 | 0.0161756 | 0.0162916 | 0.0179109 | 0.0178714 | 0.0186856 | 0.0178824 | 0.0171189 | 0.0173694 |
| 187 | 0.00645109 | 0.00593676 | 0.00598399 | 0.00648904 | 0.00624668 | 0.00663774 | 0.00648009 | 0.00638549 | 0.00617825 |
| 194 | 0.124581 | 0.122524 | 0.123555 | 0.120333 | 0.119936 | 0.128661 | 0.12052 | 0.119403 | 0.122808 |
| 197 | 0.268528 | 0.254371 | 0.256289 | 0.262437 | 0.255993 | 0.268666 | 0.262688 | 0.263971 | 0.246145 |
| 198 | 0.225338 | 0.214649 | 0.216141 | 0.223696 | 0.217531 | 0.23861 | 0.224524 | 0.22509 | 0.194012 |
| 207 | 0.163164 | 0.158013 | 0.159153 | 0.160084 | 0.157216 | 0.175884 | 0.159675 | 0.158344 | 0.150697 |
