# Supplementary material for: Insilico prediction and functional analysis of nonsynonymous SNPs in human CTLA4 gene
Source: Sci Rep. 2022 Nov 28;12:20441. doi: 10.1038/s41598-022-24699-0 (PMC9705290; doi:10.1038/s41598-022-24699-0)
Supplement: Supplementary file 1 — Supplementary Information. [file 41598_2022_24699_MOESM1_ESM.zip › Supplementary Data/Table S7.docx]

**Table S7:** CCR6 Ubiquitination Prediction Results by UbPred and BDM-PUB

| **UbPred** | | | **BDM-PUB** | | |
| --- | --- | --- | --- | --- | --- |
| **Residue** | **Score** | **Ubiquitinated** | Position | Score | Threshold |
| 10 | 0.25 | No | 10 | 0.49 | 0.3 |
| 36 | 0.45 | No | 65 | 1.08 | 0.3 |
| 65 | 0.62 | Yes | 188 | 2.45 | 0.3 |
| 130 | 0.48 | No | 191 | 1.91 | 0.3 |
| 188 | 0.35 | No | 192 | 1.97 | 0.3 |
| 191 | 0.13 | No |  |  |  |
| 192 | 0.13 | No |  |  |  |
| 203 | 0.76 | Yes |  |  |  |
| 213 | 0.62 | Yes |  |  |  |

**Legend for UbPred:**

| Label | Score range | Sensitivity | Specificity |
| --- | --- | --- | --- |
| Low confidence | 0.62 ≤ s ≤ 0.69 | 0.464 | 0.903 |
| Medium confidence | 0.69 ≤ s ≤ 0.84 | 0.346 | 0.950 |
| High confidence | 0.84 ≤ s ≤ 1.00 | 0.197 | 0.989 |
