# Supplementary material for: Insilico prediction and functional analysis of nonsynonymous SNPs in human CTLA4 gene
Source: Sci Rep. 2022 Nov 28;12:20441. doi: 10.1038/s41598-022-24699-0 (PMC9705290; doi:10.1038/s41598-022-24699-0)
Supplement: Supplementary file 1 — Supplementary Information. [file 41598_2022_24699_MOESM1_ESM.zip › Supplementary Data/Table S8.docx]

**Table S8:** Interaction of CTLA4 with other genes predicted by STRING

| Interaction genes | Combined Score | Predictions for specific actions |
| --- | --- | --- |
| CD86 | 0.999 | \| Activation: \| yes (score: 0.804) \| \| --- \| --- \| \| Binding: \| yes (score: 0.902) \| \| Expression: \| yes (score: 0.615) \| \| Reaction: \| yes (score: 0.902 \| |
| CD80 | 0.999 | \| Activation: \| yes (score: 0.818) \| \| --- \| --- \| \| Binding: \| yes (score: 0.909) \| \| Expression with Inhibition: \| yes (score: 0.615) \| \| Reaction: \| yes (score: 0.909) \| |
| FOXP3 | 0.997 | \|  \| \| --- \| \| Activation: \| yes (score: 0.900) \| \| Expression: \| yes (score: 0.565) \| \| Expression with Inhibition: \| yes (score: 0.565) \| |
| LCK | 0.989 | \| Binding: \| yes (score: 0.900) \| \| --- \| --- \| \| Catalysis: \| yes (score: 0.900) \| \| Post-translational modification: \| yes (score: 0.407) \| \| Reaction: \| yes (score: 0.900) \| |
| FYN | 0.987 | \|  \| \| --- \| \| Binding: \| yes (score: 0.907) \| \| Catalysis: \| yes (score: 0.907) \| \| Post-translational modification: \| yes (score: 0.483) \| \| Reaction: \| yes (score: 0.907) \| |
| PTPN11 | 0.982 | \| Activation: \| yes (score: 0.900) \| \| --- \| --- \| \| Binding: \| yes (score: 0.900) \| |
| ITGA4 | 0.973 | \| Binding: \| yes (score: 0.900) \| \| --- \| --- \| |
| ICOSL | 0.971 | \|  \| \| --- \| \| Activation: \| yes (score: 0.800) \| \| Binding: \| yes (score: 0.499) \| |
| ITGAL | 0.967 | \| Binding: \| yes (score: 0.900) \| \| --- \| --- \| |
| IL17A | 0.967 | \| Expression: \| yes (score: 0.800) \| \| --- \| --- \| |
